# Simulated early Earth geochemistry fuels a hydrogen-dependent primordial metabolism

**DOI:** 10.1038/s41559-025-02676-w

**Published:** 2025-04-30

**Authors:** Vanessa Helmbrecht, Robert Reichelt, Dina Grohmann, William D. Orsi

**Affiliations:** 1 Department of Earth and Environmental Sciences, Ludwig-Maximilians-Universität München, Munich, Germany; 2 Institute of Biochemistry, Genetics and Microbiology, Institute of Microbiology and Archaea Centre, Single-Molecule Biochemistry Lab and Regensburg Center for Biochemistry, University of Regensburg, Regensburg, Germany; 3 GeoBio-CenterLMU, Ludwig-Maximilians-Universität München, Munich, Germany

**Keywords:** Palaeontology, Archaea, Origin of life, Microbial ecology

## Abstract

Molecular hydrogen is the electron donor for the ancient exergonic reductive acetyl-coenzyme A pathway (acetyl-CoA pathway), which is used by hydrogenotrophic methanogenic archaea. How the presence of iron-sulfides influenced the acetyl-CoA pathway under primordial early Earth geochemistry is still poorly understood. Here we show that the iron-sulfides mackinawite (FeS) and greigite (Fe_3_S_4_), which formed in chemical garden experiments simulating geochemical conditions of the early Archaean eon (4.0–3.6 billion years ago), produce abiotic H_2_ in sufficient quantities to support hydrogenotrophic growth of the hyperthermophilic methanogen *Methanocaldococcus jannaschii*. Abiotic H_2_ from iron-sulfide formation promoted CO_2_ fixation and methanogenesis and induced overexpression of genes encoding the acetyl-CoA pathway. We demonstrate that H_2_ from iron-sulfide precipitation under simulated early Earth hydrothermal geochemistry fuels a H_2_-dependent primordial metabolism.

## Abstract (web summary)

In an iron-sulfide chemical garden experiment that mimics the kind of hydrothermal vents that might have occurred in the early Archaean, mackinawite and greigite are precipitated and abiotic H_2_ is produced in sufficient quantities to support exponential growth of a hyperthermophilic and hydrogenotrophic methanogen, *Methanocaldococcus jannaschii*.

## Main

Formation of iron-sulfide minerals is an ancient and globally widespread process known to proceed via mackinawite (FeS)^1^ and the intermediate greigite (Fe_3_S_4_) (refs. ^2,3^) while producing H_2_ (refs. ^4–7^). The ancient occurrence of hydrothermal iron-sulfide rich deposits in the geological record extend into the early Archaean Eon (Eoarchaean 4.0–3.6 billion years ago) and exhibit fossil features interpreted as some of the oldest signatures for life on Earth^8,9^. Chemical garden experiments have shown the mackinawite–greigite transition under hydrothermal conditions analogous to Eoarchaean geochemical environments^7,10^. Such iron-sulfide chemical gardens are mixtures of inorganic chemicals which often form precipitates resembling organic structures and have been applied in emergence of life studies to better understand connections between aqueous geochemistry and mineralogy in simulated hydrothermal springs^10,11^. However, links between abiotic H_2_ production in iron-sulfide chemical gardens simulating Eoarchaean hydrothermal systems and early life are scarce.

Molecular H_2_ can be produced abiotically in numerous geological environments, most notably by serpentinization involving mafic (Mg and Fe-rich) or ultramafic rocks^12,13^. Since their discovery in 1977^14^, seafloor hydrothermal springs venting abiotic H_2_ were viewed as potential energy sources and setting for the emergence of life^15–19^. Reactive iron-sulfide minerals are ubiquitous in hydrothermal vent black-smoker chimneys, which are surrounded by metalliferous sediments underlying the hydrothermal plumes of precipitated black iron-sulfide particles^15,20,21^. Modern black smokers can exhibit natural variations in fluid chemistry and temperature within a single vent system^22^, providing temperature gradients and sources of chemical energy^15,16,21^ to support a high diversity of microorganisms, including methanogenic archaea^20^. The availability of H_2_ plays a key role as the main source of electrons for biological systems and thereby microbial growth at hydrothermal systems^23,24^.

Models of early metabolism in the Eoarchaean predict that abiotic H_2_ was a potentially important electron donor and CO_2_ served as a key electron acceptor for the first cells^25–27^. Anaerobic organisms that use the H_2_-dependent reductive acetyl-coenzyme A (acetyl-CoA) pathway for CO_2_ fixation, such as methanogens and acetogens, are modern representatives that have preserved vestiges of the first metabolisms^28,29^. Geochemical evidence has shown that methanogenic microbes have been present since the early Archaean at least 3.5 billion years ago^29^, and it has been proposed that methanogens could have emerged in an iron-sulfide hydrothermal black-smoker environment^16^. Furthermore, physiological predictions of the last universal common ancestor (LUCA) based on genome data indicated that LUCA was possibly (hyper)thermophilic^30^, anaerobic and H_2_-dependent^31^, and used the acetyl-CoA pathway to fix CO_2_ (refs. ^27,31–34^). The acetyl-CoA pathway is the most ancient among carbon-fixation pathways^35^, as it is short, non-cyclic, exergonic^33,35,36^ and the only carbon-fixation pathway that can be reconstituted in the laboratory to take place without enzymes^25,37^.

The acetyl-CoA pathway is furthermore replete with enzymes that depend on simple Fe(Ni)S cofactors^27,31,33,36,38^. For example, the CO_2_ reducing-and-fixing enzyme formylmethanofuran dehydrogenase in methanogens contains an anomalously high 46 [4Fe–4S] clusters^39^. Other enzymes such as ferredoxin and CO dehydrogenase, which are common to methanogens, have iron-sulfur clusters with similar structure to iron-sulfide minerals, such as mackinawite and greigite^18,36^, which form in hydrothermal environments. These observations have led to the hypothesis that iron-sulfide clusters in these modern enzymes might be relics of an ancient metabolism, which possibly originated in an ancient iron-sulfide rich setting^31,40^.

These data and observations suggest an ancient origin of the acetyl-CoA pathway as a potential primordial metabolism^25,32–35,41^. Because of the possibility that hyperthermophilic methanogens harbour one of the most ancient metabolisms^30^, we investigated acetyl-CoA pathway-dependent growth of the methanogenic hyperthermophile *Methanocaldococcus jannaschii* during iron-sulfide mineral formation.

Ferruginous (anoxic and iron-rich^42^) Archaean oceans contained dissolved ferrous iron concentrations of 0.1–10 mM (refs. ^43,44^). Sulfide may have been relatively rare as shown by its apparent dearth in the Hadean and Eoarchaean, but the origin of sulfide could have been derived from volcanic sulfur dioxide and polymerized sulfides^45,46^. In the absence of sulfidic fluids, green rust was probably a major constituent of Hadean and Archaean chemical gardens under ferruginous conditions^47,48^. Sulfate was extremely rare in the Archaean oceans^49^, and the salinity has been predicted to be similar^50^ if not greater^51^ than now found in ocean water. We attempted to reflect these ancient conditions of the Eoarchaean oceans in our chemical gardens (Supplementary Methods).

As our model organism, we chose the methanogenic hyperthermophile *M. jannaschii* (DSM strain 2661), which was originally isolated from hydrothermal iron-sulfide sediment at the base of a black smoker on the East Pacific Rise^52^. *M. jannaschii* serves as a model organism for methanogenesis^53–55^; it uses H_2_ and CO_2_ as its sole carbon and energy source and has an optimal growth temperature ~85 °C (refs. ^52^). Thus, tracing both H_2_ and CO_2_ and the product CH_4_ can reveal the active metabolism of this organism in controlled experiments, which is advantageous, because only a small amount of substrates and products need to be measured to study the energy metabolism.

## Results and discussion

### Iron-sulfide chimney formation, mineralogy and habitability

To precipitate iron-sulfide, we created sulfidic precipitation mounds (experiment 1, Supplementary Table 1) within a ferruginous aqueous environment germane to hydrochemical conditions predicted for the Hadean Ocean at the time of the origin of life^7,10,18^. Acidic sulfidic solution (0.5 M Na_2_S, pH 3) was injected into an amorphous ferruginous solution (0.5 M Fe(II)Cl_2_, pH 6) (chimney protocol in Supplementary Methods). Within 10 min, iron-sulfide minerals crystallized forming a black-chimney structure reaching an average height of 2.0 cm (s.d. = 0.1). In this initial phase of the experiment, the chimney showed the most rapid growth (growth rate = 0.2 cm min^−1^). After 1 hour, the average height of the chimney was 2.5 cm (s.d. = 0.1, growth rate = 0.01 cm min^−1^). No growth of the chimney was observed after 1 hour. All experiments were performed under an anoxic N_2_ atmosphere in an anoxic chamber. Additional information about imaging, processing and quantifying the chimney growth is displayed in the Supplementary Methods.

The chimneys were stable at room temperature. Upon heating of the injection fluid, however, bubbles formed in the sulfidic fluid and caused the chimney structures to collapse into sediment, which collected at the bottom of the flask. Therefore, all hydrothermal experiments at 80 °C were conducted using sedimented iron-sulfide material (for details of preparation, see experiments 2, 3, 4 and 5 in Supplementary Table 1), which we describe as ‘sedimentary iron-sulfide chemical gardens’. Future experiments could test if higher pressure (similar to the deep sea) would increase the boiling temperature of the injected sulfidic fluid and reduce formation of gas bubbles.

**Fig. 1:**
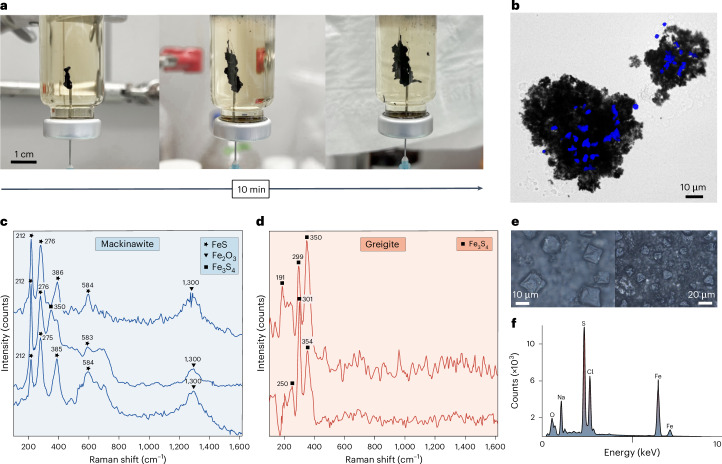
Iron-sulfide chimney formation, mineralogy and microbial colonization. **a**, Iron-sulfide chimney precipitated over the course of 10 min at 25 °C (experiment 1, Supplementary Table 1). The three images (from left to right) were taken 1, 5 and 10 min after the injection started. After 10 min, the chimney reached a height of 2.0 cm (s.d. = 0.1). **b**, *M. jannaschii* cells accumulate on iron-sulfide particles (experiment 4a). Overlay of a bright field image of the iron-sulfide particles and a fluorescence image of the cells. This was observed across three experimental replicates (Methods). **c**–**f**, Mineralogical analysis (experiment 5a). **c**,**d**, Raman spectroscopy identifies that mackinawite (FeS, **c**) and greigite (Fe_3_S_4_, **d**) are the primary minerals formed in the sedimentary iron-sulfide chemical gardens at 80 °C. This was observed across three experimental replicates (Supplementary Table 1). Important Raman peaks are labelled for mackinawite and greigite^7,57–59^. The peak at 1,300 cm^−1^ is due to laser beam oxidation. **e**, Raman images of a sedimentary iron-sulfide chemical garden. Most crystals have an octahedral shape typical for greigite. **f**, EDX of iron-sulfide particles with peaks indicating relative abundances of O, Na, S, Cl and Fe. keV, kiloelectron volt.

Raman spectroscopy and scanning electron microscopy (SEM) revealed that the hydrothermal (80 °C) sedimentary iron-sulfide chemical gardens (experiment 5a, Supplementary Table 1) comprised mostly mackinawite (FeS) and greigite (Fe_3_S_4_), with traces of NaCl (Fig. 1c–f). Under anoxic conditions, metastable mackinawite, an iron-monosulfide precursor, transforms into greigite when heated to 70–75 °C through partial oxidation of mackinawite with water^2,7^.

**Extended Data Fig. 1:**
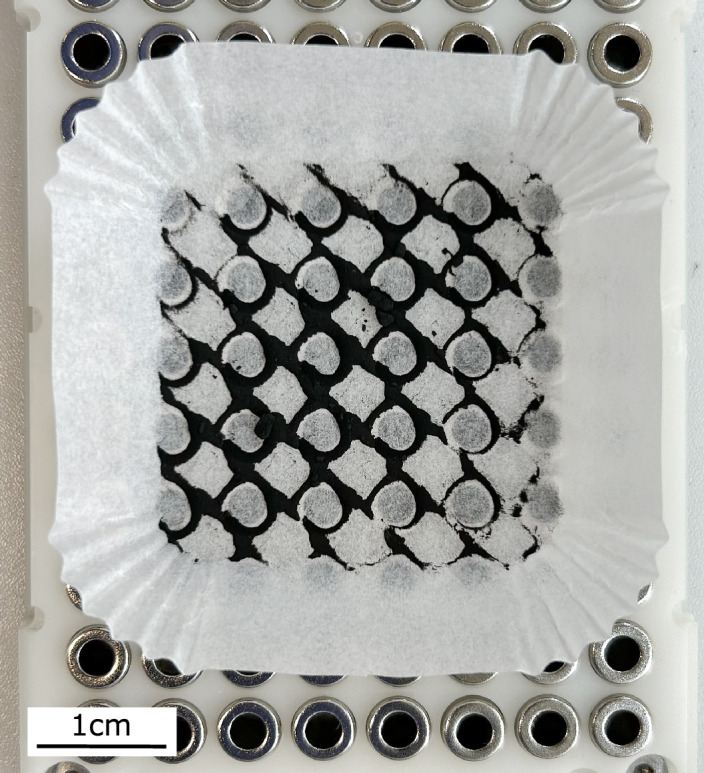
Iron-sulfide particles are magnetic. Sedimentary iron-sulfide chemical garden precipitates align with magnetic rings on a ring magnet plate, showing the presence of the magnetic iron-sulfide mineral greigite (Fe_3_S_4_).

Mackinawite is a tetragonal iron-monosulfide^1^. Greigite is the cubic thio-spinel of iron, exhibiting mostly octahedral and sometimes cubic crystals^2,3,56^. Next to the Raman peaks for mackinawite at 212, 275–276, 385–386 and 583–584 cm^−1^ (Fig. 1c)^57,58^ a broad peak at 1,300 cm^−1^ was evident of slight laser beam oxidation of the sample, which converted mackinawite into haematite (Fe_2_O_3_). The greigite Raman spectra (Fig. 1d) show peaks at 191, 250, 299–301 and 350–354 cm^−1^ (refs. ^7,59^). The Raman images display an octahedral crystal morphology fitting for greigite^3^ (Fig. 1e). The energy dispersive X-ray (EDX) spectrum highlights the presence of iron (30.78 at.%) and sulfur (41.33 at.%) in the sample (Fig. 1f). Magnetic properties of the iron-sulfide minerals from the chemical garden (experiment 5a, Supplementary Table 1) was confirmed with a 96-ring magnet plate (ALPAQUA) (Extended Data Fig. 1), indicating the presence of greigite, which is a ferrimagnetic mineral^2,56,60,61^. These results are consistent with earlier chemical garden experiments^7^, which also produced semipermeable colloidal membranes of iron-monosulfide minerals mimicking natural hydrothermal chimneys^10^.

Iron-sulfur clusters play a critical role in various metalloenzymes that are present in both archaea and bacteria^7^, and therefore mackinawite and greigite are considered key minerals for the emergence of life^7,40,59,62^ because they structurally resemble active centres of proteins and enzymes^36,59,63,64^. The presence of both minerals in hydrothermal environments hints at the possibility that iron-rich cofactors in proteins and enzymes geologically originate from mineral precursors^63^. It is therefore notable that mackinawite and greigite are the major precipitated minerals in our chemical gardens simulating sulfidic Eoarchaean hydrothermal springs (Fig. 1d). We note that the pyrite structure is different because the ferrous iron is ligated to six sulfur pairs, which makes the mineral less reactive by comparison^65^.

To test the habitability of the sedimentary iron-sulfide chemical gardens, we added stationary-phase culture of the hyperthermophilic methanogen *M. jannaschii*^52^ to the chemical gardens (experiment 4a, Supplementary Table 1). Fluorescence microscopy revealed *M. jannaschii* cells physically associated with iron-sulfide particles (Fig. 1b), consistent with minerals found in black smokers that are colonized by thermophilic archaea^66,67^. The pH of the sedimentary chemical gardens was 5.5, which is close to the optimal pH for growth of *M. jannaschii*^68^. Next, we investigated mechanisms linking the mineralogy of the iron-sulfide chemical gardens to microbial habitability.

### Abiotic H_2_ production and methanogenesis in chemical gardens

**Fig. 2:**
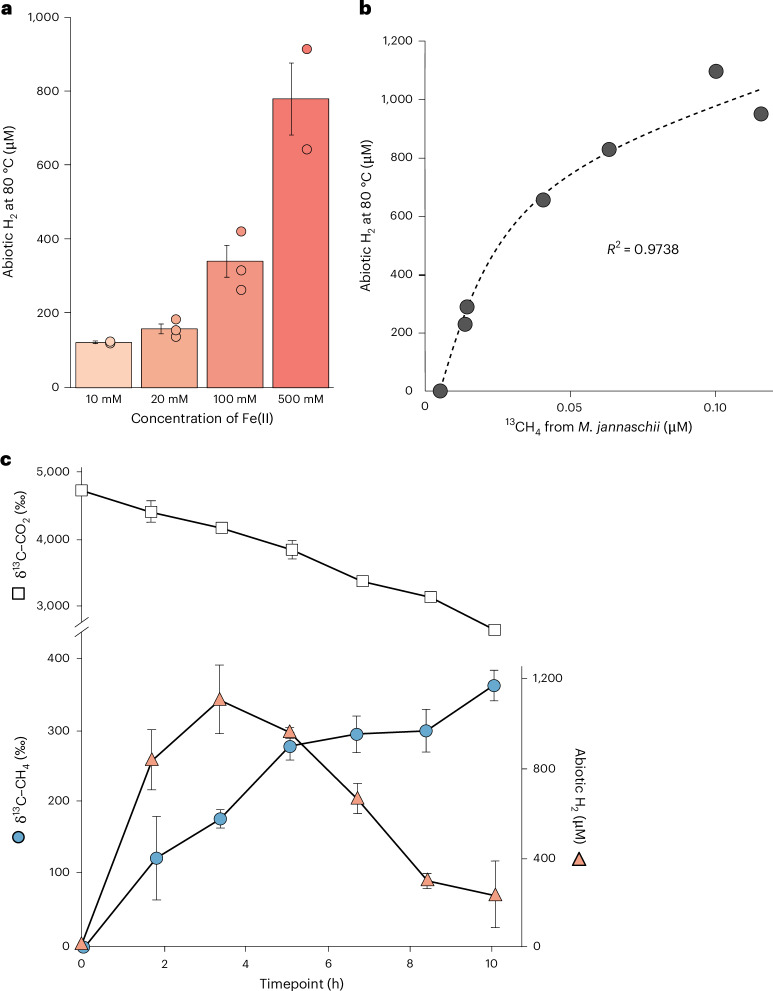
Abiotic H_2_ produced by the iron-sulfide chemical garden fuels CO_2_ reduction and methanogenesis by *M. jannaschii.* **a**, Production of abiotic H_2_ in the chemical gardens increases with increasing Fe(II) concentrations at 80 °C (experiment 2, Supplementary Table 1). Error bars indicate standard error of the means, individual points represent biological replicates (*n* = 3). **b**, The concentration of abiotic H_2_ produced in the sedimentary iron-sulfide chemical garden (500 mM FeCl_2_ and Na_2_S, 80 °C) correlates positively with the amount of ^13^CH_4_ produced by *M. jannaschii*. **c**, Gas consumption and production by *M. jannaschii* in the sedimentary iron-sulfide chemical gardens at 80 °C supplemented with ^13^C-bicarbonate to trace CO_2_ fixation and methanogenesis (experiment 3). Error bars represent standard deviations across three biological replicates. Note that the ^13^C-labelling of CO_2_ decreases over time, as the ^13^C-labelling of methane increases, indicating a transfer of ^13^C from CO_2_ to CH_4_ by *M. jannaschii*. The abiotically produced H_2_ increases over the first 4 h and then decreases afterwards, due to consumption by *M. jannaschii*.

We detected hydrogen gas (H_2_) emanating from the sedimentary iron-sulfide chemical gardens (experiment 2, Supplementary Table 1) in amounts proportional to the concentrations of Fe(II) and Na_2_S (Fig. 2a). Abiotic H_2_ was only detectable in flasks where no headspace was present (Supplementary Methods), which could be due to increasing pressure building without a larger headspace volume. The concentration of abiotic H_2_ produced in the chemical gardens ranged from 124 ± 1 to 781 ± 138 µM, spanning Fe(II) and Na_2_S concentrations of 10–500 mM, respectively (*P* = 0.0206) (Fig. 2a). This is in the range of H_2_ concentrations in the fluids of black smokers, from 0.05 to 1 mM (ref. ^12^). Abiotic H_2_ production is probably explained by the presence of mackinawite (FeS) and greigite (Fe_3_S_4_) above 70 °C (Fig. 1c,d), namely that H_2_ is released during the hydration of iron-monosulfide^6,7,69^ according to:

$$4{\rm{FeS}}+{2{\rm{H}}}_{2}{\rm{O}}\to {{\rm{Fe}}}_{3}{{\rm{S}}}_{4}+{{\rm{Fe}}({\rm{OH}})}_{2}+{{\rm{H}}}_{2}$$

**Extended Data Fig. 2:**
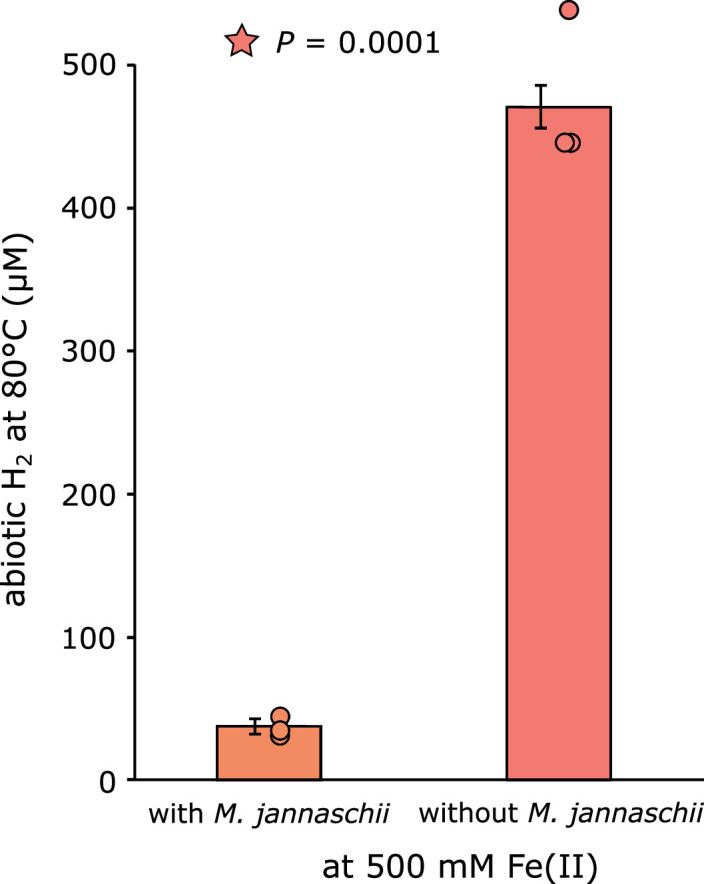
Abiotic H_2_ produced in the 500 mM sedimentary iron-sulfide chemical gardens at 80 °C with and without *M. jannaschii.* Error bars show standard error of the means across three biological replicates, P value is the result of a one-sided T-test.

A similar reaction of mackinawite and H_2_S was found to be used in an anaerobic metabolism by H_2_-producing bacteria in a syntrophic partnership with hydrogenotrophic methanogens^70^. Addition of stationary-phase *M. jannaschii* culture to the chemical garden reduced abiotic H_2_ significantly compared to controls (*P* = 0.0001) without added methanogens (Extended Data Fig. 2), indicating that the hydrogenotrophic *M. jannaschii* cells consume H_2_ for growth. Others^54^ defined the lower limit for growth at 17–23 µM of H_2_, which is less H_2_ than the iron-sulfide chemical gardens produced (experiment 2, Fig. 2a). No H_2_ was detectable in controls where FeCl_2_ and Na_2_S were not added. We investigated the effects of abiotic H_2_ from the sedimentary chemical gardens on the physiology of *M. jannaschii* (experiment 3, Fig. 2c) by tracking the production of H_2_ and ^13^CH_4_ at 80 °C (experiment 3, Supplementary Table 1) using ^13^CO_2_ carbon substrate. After inoculation, an increase in ^13^CH_4_ was observed concomitant with a decrease in ^13^CO_2_, as expected for *M. jannaschii* methanogenesis (Fig. 2c). Over the course of 10 h, ^13^C-labelled CO_2_ decreased constantly, while abiotic H_2_ increased in concentration until 4 h, after which the rate of H_2_ consumption by *M. jannaschii* began to outpace H_2_ production. We observed a direct correlation (*R*^2^ = 0.97, *P* < 0.005) between abiotic H_2_ produced by the chemical garden and ^13^C-labelled CH_4_ produced by *M. jannaschii* (Fig. 2b) indicating that, consistent with earlier studies^54,55^, hydrogenotrophic methanogenesis by *M. jannaschii* in the iron-sulfide chemical gardens was limited by H_2_.

### *M. jannaschii* growth in iron-sulfide chemical gardens

We compared the growth of *M. jannaschii* at 80 °C in the iron-sulfide chemical garden (experiment 5a) to growth in MMC medium (experiment 5c) as a positive control and sterile water (experiment 5b) as a negative control. Similar to experiment 4, the sterile water negative control was used to account for the dilution factor introduced by inoculating the stationary-phase culture into the chemical garden, making the presence of iron-sulfide the only difference between experiments 5a and 5b (Supplementary Table 1).

**Fig. 3:**
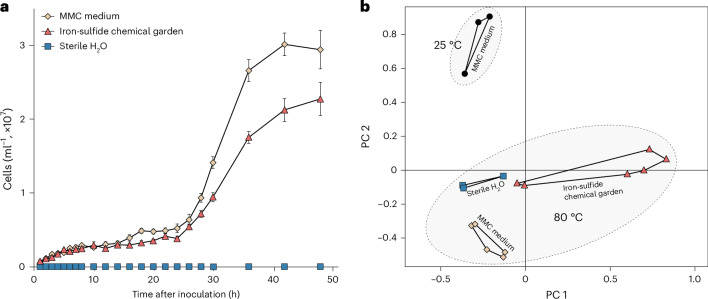
*M. jannaschii* growth and gene expression in the sedimentary iron-sulfide chemical gardens compared to controls. **a**, Comparison of *M. jannaschii* growth in three experimental settings: sedimentary iron-sulfide chemical gardens (experiment 4a, Supplementary Table 1), MMC growth medium (experiment 4c) and sterile water (experiment 4b). All experiments were performed at 80 °C, error bars indicate standard error of the means across three biological replicates. **b**, Principal-component (PC) analysis shows the significant difference of *M. jannaschii* gene expression in non-identical treatments (experiment 5) (analysis of similarity *P* = 0.001). Grey circles highlight transcriptomes from 25 and 80 °C experiments, respectively. Coloured symbols correspond to the same three experimental conditions that are displayed in **a** (yellow diamonds, MMC media control; orange triangles, iron-sulfide chemical garden; blue squares, sterile water negative control).

*M. jannaschii* reached exponential growth (growth rate *µ* = 0.14 h^−1^) in the iron-sulfide chemical gardens after 24 h reaching a peak cell concentration of 2.3 × 10^7^ cells ml^−1^ (Fig. 3a) (experiment 4a, Supplementary Table 1). However, the growth rate in the chemical gardens (experiment 4a) was 30% lower (*P* = 0.0424) than the positive controls (*µ* = 0.20 h^−1^), where *M. jannaschii* was grown in their standard MMC growth medium (experiment 4c) and reached a peak cell concentration of 3.0 × 10^7^ cells ml^−1^, lower than previous reports^52^ possibly because we did not shake cultures during incubation. The lower growth rate in the iron-sulfide chemical gardens (Fig. 3a) could be explained by the lack of additional salts, trace metals (for example, Ni, Mo, Co, W and Zn) and nutrients compared to MMC medium, where nitrogen in the form of ammonia was present at tenfold higher concentrations^68^. Traces of nitrogen and other essential metals were inevitably transferred with the inoculum to the chemical gardens and were probably available—albeit at ultra-low concentrations—for growth (Fig. 3a).

No growth was observed in the negative (water) control (experiment 4b) (Fig. 3a). Because the addition of Fe(II)Cl_2_ and Na_2_S are the only parameters that differed compared to the control (additional nutrients, vitamins or trace metals above those present in the inoculum were not added to the chemical gardens), exponential growth in the chemical gardens can be attributed to H_2_ from the reaction of FeCl_2_ and Na_2_S (Fig. 2c and Extended Data Fig. 2). Nitrogen limitation cannot explain the large difference in growth rate between the water control and the chemical garden, because both experiments received the same amount of inoculum. This indicates that the growth in the chemical gardens was mainly limited by H_2_ compared to nitrogen, indicating that the abiotic H_2_ from the chemical garden was sufficient enough to rescue *M. jannaschii* from H_2_ limitation.

### Gene expression in iron-sulfide chemical gardens

RNA was extracted from the 80 °C sterile water control (experiment 5b), 10 mM iron-sulfide chemical garden (experiment 5a) and MMC medium (experiment 5c), which was determined to be 2.52, 2.90 and 10.00 ng µl^−1^, respectively (Supplementary Methods). This RNA was used for transcriptomic analysis of *M. jannaschii*, which showed that gene expression was significantly different between the three 80 °C treatments (10 mM iron-sulfide, MMC medium and sterile water, experiments 5a–c, Supplementary Table 1), indicating that each condition was associated with different physiological states (Fig. 3b). The sedimentary iron-sulfide chemical garden transcriptomes uncovered biological (analysis of similarity *P* = 0.001) and technical variation in between replicates exceeding that in controls (Fig. 3b), but nonetheless show a reproducible and unique gene expression profile induced by iron-sulfide chemical gardens. Key genes that were overexpressed by *M. jannaschii* in the chemical gardens reflect the H_2_-dependent nature of *M. jannaschii* metabolism.

**Fig. 4:**
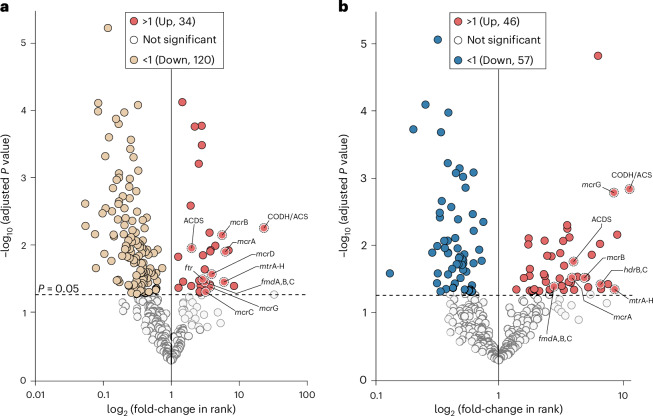
Volcano plot analysis of gene expression of *M. jannaschii* transcriptomes in the iron-sulfide chemical gardens. Gene expression is compared to two sets of controls (experiments 5a–c, Supplementary Table 1). **a**, Significant overexpression of genes (*n* = 34, *P* < 0.05) in *M. jannaschii* transcriptomes from 80 °C iron-sulfide chemical gardens (red, experiment 5a) relative to the MMC medium (beige, experiment 5c) (*P* = 0.05, one-sided *t*-test). **b**, Significant overexpression of genes (*n* = 46, *P* < 0.05) in *M. jannaschii* transcriptomes from 80 °C iron-sulfide chemical gardens (red, experiment 5a) relative to sterile water negative controls (blue, experiment 5b) (*P* = 0.05, one-sided *t*-test). The vertical line separates expressed genes that either increased or decreased in rank in the chemical gardens, the dotted horizontal line represents the *P* value cutoff for determining statistical significance. Overexpressed genes in the chemical gardens that are part of the methanogenic acetyl-CoA pathway are labelled. The full spellings of the gene acronyms of the acetyl-CoA pathway are provided in Supplementary Tables 1 and 2. Note that the genes of the methanogenic acetyl-CoA pathway were only overexpressed in the iron-sulfide chemical gardens, and were overexpressed relative to the MMC medium and sterile water negative controls.

**Extended Data Fig. 3:**
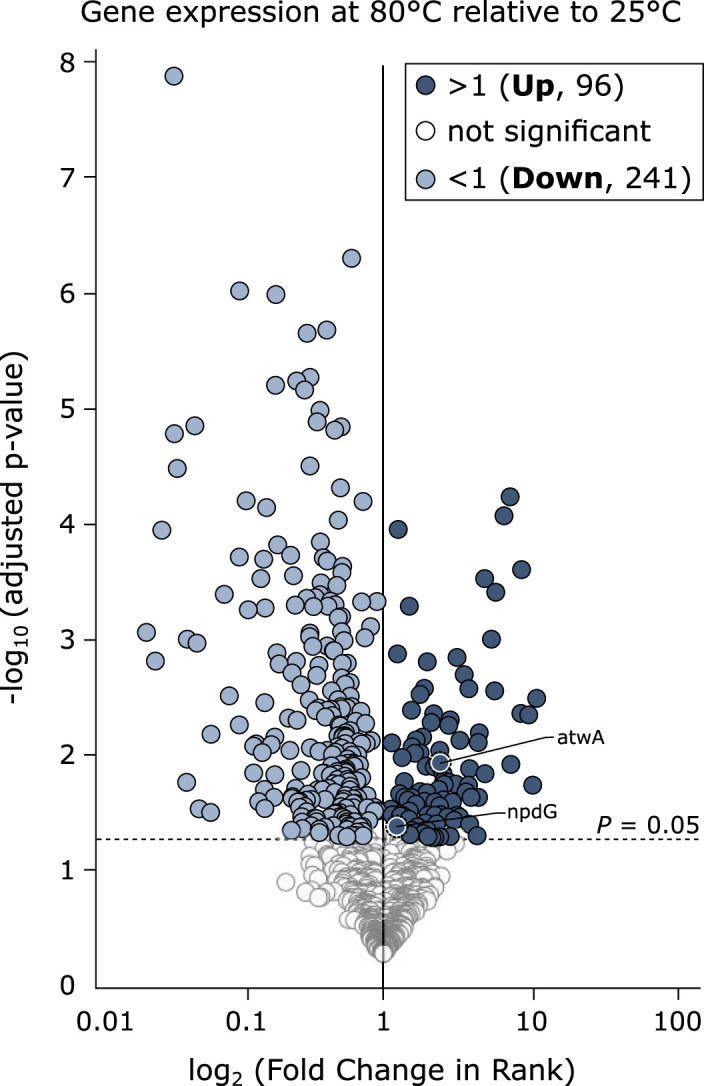
Volcano plot analysis of gene expression of *M. jannaschii* transcriptomes in MMC Medium at 25 and 80 °C (*Experiments 5c, d*, Supplemental Table 1). Of all significant genes (*P* = 0.05, one-sided t-test), 96 are overexpressed at 80 °C (dark blue, *Experiment 5c*) compared to 25 °C. 241 genes were down regulated at 80 °C compared to 25 °C (light blue, *Experiment 5* *d*). The vertical line separates expressed genes that either increased or decreased in rank in the MMC medium, the dotted horizontal line represents the *P*-value cutoff for determining statistical significance (one-sided T-test). Of all overexpressed genes, two are part of the methanogenic acetyl CoA pathway: methyl coenzyme M reductase system component A2 (*atw*A) and NADPH-dependent F420 reductase (*npd*G).

In contrast to the MMC medium, 120 genes were downregulated in *M. jannaschii* grown in sedimentary iron-sulfide chemical gardens, whereas 57 genes were downregulated in *M. jannaschii* cells grown in chemical gardens compared to the water controls (experiment 5, Fig.  4 and Supplementary Table 1). However, numerous genes encoding key enzymes of the H_2_-dependent and CO_2_-fixing acetyl-CoA pathway for methanogenesis were overexpressed in cells grown in chemical gardens when compared to the controls (Fig. 4 and Supplementary Tables 2 and 3). A total of 34 and 46 genes were overexpressed in cells grown in chemical gardens as compared to MMC medium (Fig.  4a and Supplementary Table 2) and water (Fig.  4b and Supplementary Table 3), respectively. The conditions of the chemical garden promoted the expression of the acetyl-CoA pathway in *M. jannaschii* compared to other cellular processes. There were 96 genes upregulated and 241 genes downregulated in *M. jannaschii* cells grown in MMC medium at the optimal 80 °C compared to MMC media at 25 °C (Extended Data Fig. 3).

The overexpressed genes of the methanogenic acetyl-CoA pathway (Fig. 4 and Supplementary Tables 2 and 3) consisted of CO dehydrogenase/CO-methylating acetyl-CoA synthase complex subunit beta (CODH/ACS), methyl-coenzyme M (methyl-COM) reductase subunits alpha/beta/gamma (*mcrA, mcrB* and *mcrG*), methyl-COM reductase operon proteins C/D (*mcrC* and *mcrD*), tetrahydromethanopterin *S*-methyltransferase subunits A–H (*mtr*A–H), formylmethanofuran dehydrogenase (*fmdA, fmdB* and *fmdC*), formylmethanofuran-tetrahydromethanopterin *N*-formyltransferase (*ftr*), acetyl-CoA decarbonylase/synthase complex subunits alpha/beta/gamma (ACDS) and coenzyme B–coenzyme M (CoB–CoM) heterodisulfide reductase subunits B/C (*hdrB* and *hdrC*). With the exception of only four genes (*mcrC*, *mcrD*, *ftr* and *hdrB,C*) all other acetyl-CoA pathway genes were overexpressed in the sedimentary iron-sulfide chemical gardens compared to both sterile water and MMC medium controls (Fig. 4). This shows that the chemical gardens were generally associated with a stimulated activity of the acetyl-CoA pathway in *M. jannaschii*, even compared to the growth conditions of the MMC medium, which was optimized for growth of hyperthermophilic methanogens^66^ (Fig. 3a).

Methanogenesis requires several carbon carriers for the reduction of CO_2_ to CH_4_, which are the coenzymes methanofuran, tetrahydromethanopterin and CoM^18,71^. CoB is not a carbon carrier but is also required in the terminal step of methanogenesis^71^. Next to methane production through CO_2_ fixation, the acetyl-CoA pathway also produces the metabolic intermediate acetyl-CoA, which is for example a precursor to lipids and pyruvate^72^. The key multi-enzyme complex involved in the acetyl-CoA formation is CODH/ACS^36,41^ where CODH catalyses the CO_2_ reduction to CO and ACS catalyses the C–C bond formation from CO and a methyl group to form acetyl-CoA^73^. This process allows methanogens to use CO as a carbon source. The multi-enzyme complex ACDS, which was also overexpressed in our sedimentary iron-sulfide chemical gardens (Fig. 4), catalyses the reversible cleavage of acetyl-CoA in methanogens^74^. Electrons for the methanogenic acetyl-CoA pathway primarily come from H_2_ (ref. ^71^). Considering that the genes responsible for the enzymatic conversions for most of these steps were overexpressed in the iron-sulfide chemical gardens, it appears that this chemolithoautotrophic pathway in *M. jannaschii* was probably driven by the abiotic H_2_ released during the formation of mackinawite and greigite in the chemical gardens.

**Extended Data Fig. 4:**
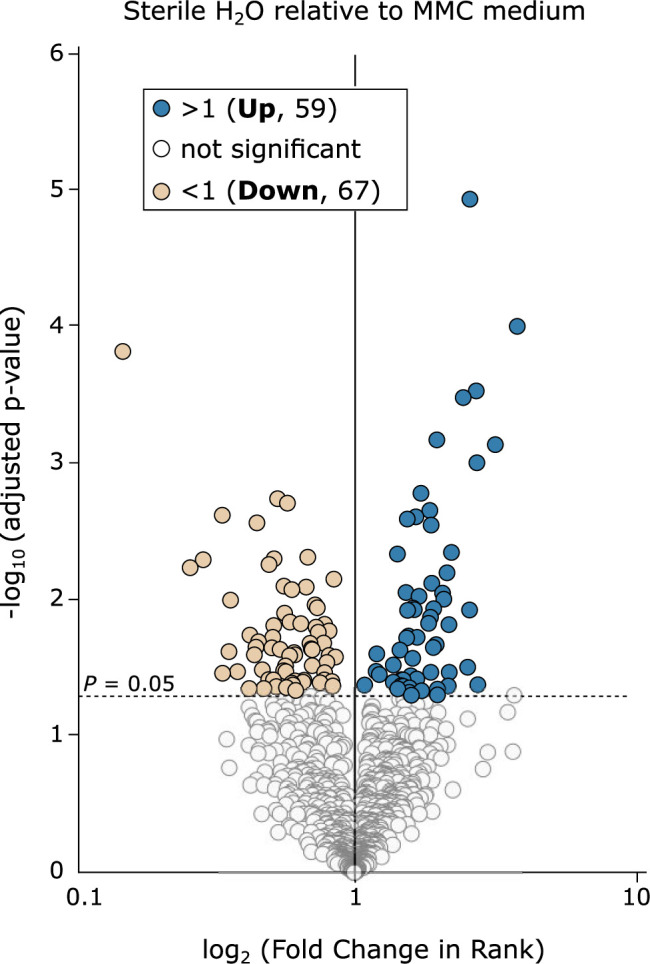
Volcano plot analysis of gene expression of *M. jannaschii* transcriptomes in sterile water relative to MMC medium (*Experiments 5b, c*, Supplemental Table 1). Of all significant genes (*P* = 0.05, one-sided t-test), 59 are overexpressed in the sterile water controls (blue, *Experiment 5b*) compared to MMC medium (beige, *Experiment 5c*). Significantly overexpressed genes are listed in Supplemental Table 4. The full list of overexpressed genes is provided in Supplemental Table 4.

Interestingly, under the non-growing physiological state in sterile H_2_O we observed an overexpression of some genes that encode enzymes of the acetyl-CoA pathway when compared to growth in MMC medium only (Extended Data Fig. 4 and Supplementary Table 4). This might indicate that non-growing archaeal cells experiencing H_2_ limitation and/or nutrient limitation can maintain a physiological readiness to quickly react to suddenly appearing favourable growth conditions in a hydrothermal environment^66,75^.

The key enzyme complex CODH/ACS (Fig. 4), which is necessary to make cell carbon^18^, contains metal sulfur clusters affine with structure iron-sulfide minerals including greigite^36,64^. Although CODH/ACS was overexpressed when cultivated in sterile H_2_O controls compared to MMC medium (Extended Data Fig. 4), the overexpression was six times higher in the chemical gardens (Fig. 4) by comparison. Specifically, CODH/ACS transcript levels had a fold-increase of 11.6 (one-sided *t*-test, *P* = 0.0014) in the iron-sulfide chemical garden relative to the sterile H_2_O, compared to 1.9-fold increase (one-sided *t*-test, *P* = 0.0007) in sterile H_2_O compared to MMC medium. This shows that while CODH/ACS is slightly overexpressed in a non-growing state, once the cells are placed into the iron-sulfide chemical garden, where they are alleviated from H_2_ limitation, they increase the expression of CODH/ACS even more as well as other genes of the acetyl-CoA pathway. This further supports the conclusion that the cells remain physiologically ready for growth even if the cells are temporarily in a non-growing state.

Overexpression of *fmd*, *ftr* and *mtr*A–H in the iron-sulfide chemical gardens is notable considering that they are part of the first CO_2_-reduction steps that take place in the acetyl-CoA pathway^71^. This is consistent with the stable isotope labelling experiment showing fixation of ^13^CO_2_ by *M. jannaschii* in the iron-sulfide chemical gardens (Fig. 2c). Interestingly, *mtr*A–H, *fmd* and *ftr* were only overexpressed in cells cultivated in chemical gardens (Fig. 4), but not in the sterile water controls compared to MMC medium (Extended Data Fig. 4 and Supplementary Table 4), indicating an enhancement of the CO_2_-reduction step by iron-sulfide conditions. This suggests that the iron-sulfide chemical garden promoted carbon fixation by *M. jannaschii*, which is consistent with the exponential growth observed in the iron-sulfide chemical gardens (Fig. 3a). No additional bicarbonate was added to the iron-sulfide chemical gardens and therefore some of the overexpression of *fmd*, *ftr* or *mtr*A–H relative to MMC medium could be a response to lower CO_2_ levels and carbon limitation. However, this cannot explain the overexpression of genes in these cells compared to the sterile H_2_O controls (because they also did not receive additional bicarbonate). Thus, it seems likely that the iron-sulfide chemical garden is a major factor influencing the overexpression of these genes.

Mcr catalyses the reduction of methyl-CoM with CoB to methane in the last step of the acetyl-CoA pathway, which is coupled to the formation of the heterodisulfide (CoM–S–S–CoB)^32,76,77^. Genes encoding all subunits of the mcr protein were overexpressed in the iron-sulfide chemical gardens relative to MMC medium and sterile H_2_O controls (Fig. 4 and Supplementary Tables 2 and 3). In contrast, only the beta subunit was overexpressed in the sterile H_2_O control compared to MMC medium and its expression level was comparably lower (Supplementary Table 4). We also detected overexpression of two *mcr* operon proteins D and G in the iron-sulfide chemical garden relative to MMC medium.

**Extended Data Fig. 5:**
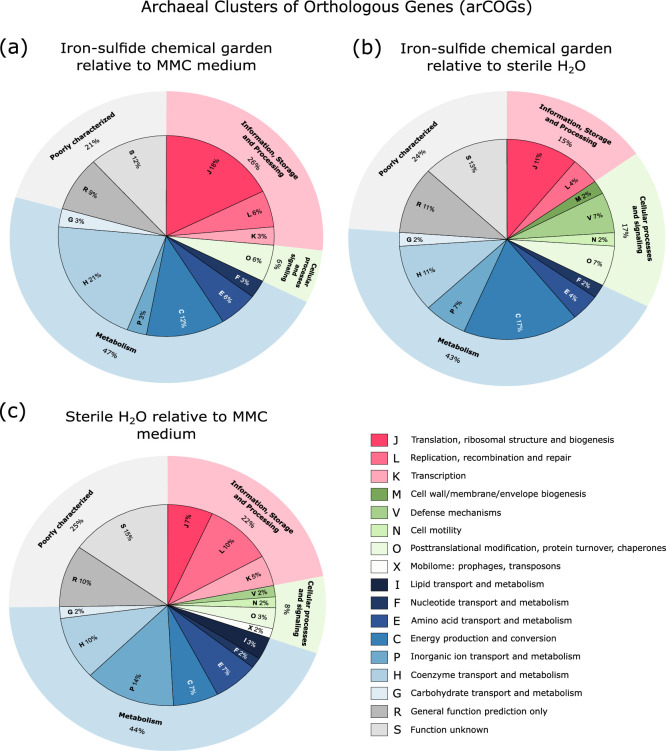
Functional annotation of overexpressed genes. (*Experiments 5a-c*, Supplemental Table 1). For gene analysis, the arCOGs (Archaeal Clusters of Orthologous Genes) database was used^79^. (**a**) Out of 34 overexpressed genes in the sedimentary iron-sulfide chemical garden relative to MMC medium (Fig. 4a), the majority of genes has a function in metabolism (blue) or information, storage and processing (red), especially in coenzyme transport (H) and translation (J). (**b**) Out of 46 overexpressed genes in the sedimentary iron-sulfide chemical garden relative to sterile H_2_O, most genes have a function in metabolism (blue), especially in energy production and conversion (C). Genes regulating cellular processes and signaling (green) make a proportion of 17 % of the overexpressed genes. (**c**) In the sterile H_2_O controls relative to MMC medium, 59 genes were overexpressed, with 44 % having a metabolic function (blue).

The heterodisulfide reductase gene (*hdr*) was only overexpressed in an active growing state compared to a non-active growing state (iron-sulfide chemical garden relative to sterile H_2_O, Fig. 4b). It was not overexpressed when comparing actively growing states to one another, specifically comparing the iron-sulfide chemical gardens to the MMC medium (Fig. 4a). This shows that hdr was important during active growth and not during dormancy. Hdr is an important iron-sulfur protein in the acetyl-CoA pathway that catalyses the reversible reduction of the heterodisulfide (CoM–S–S–CoB) back to CoM–SH and CoB–SH with H_2_ leading to the formation of methane^76,78^. The overexpression of these acetyl-CoA pathway genes *mcr* and *hdr* is furthermore consistent with the stable isotope labelling of ^13^CH_4_ which was produced by *M. jannaschii* in the iron-sulfide chemical gardens (Fig. 2c). *M. jannaschii* overexpressed a disproportionally higher percentage of genes assigned to archaeal clusters of orthologous genes (arCOGs)^79^ involved in energy production and conversion and translation in iron-sulfide chemical gardens compared to the controls (Extended Data Fig. 5). All these results indicate that cellular control of the acetyl-CoA pathway responsible for energy conservation and CO_2_ fixation was overexpressed in the chemical gardens (Fig. 4a). These experimental results support metabolic and evolutionary models based on phylogenomic reconstructions of LUCA, indicating that the acetyl-CoA pathway was present in LUCA, which might have lived in a hydrothermal environment^31,34^.

Furthermore, it has been determined that reduced iron can promote electron transfer from H_2_ to ferredoxin, which might have been important for cellular energy metabolism via the acetyl-CoA pathway before the emergence of enzymes and cofactors^80^. It therefore seems possible that reduced iron-sulfides could also facilitate electron transfer between H_2_ and ferredoxin in *M. jannaschii*, which would help to explain how *M. jannaschii* can achieve exponential growth in the iron-sulfide chemical garden.

We compared the effect of temperature to iron-sulfide environments as a factor that might influence the overexpression of genes involved in the acetyl-CoA pathway. Gene expression of *M. jannaschii* at 80 and 25 °C (experiments 5c,d and Supplementary Table 1) in MMC medium was different (Fig. 3b). Temperature is known to have a strong effect on the gene expression of thermophilic archaea^75^. In our experiments, a total of 96 genes were overexpressed at 80 °C compared to 25 °C (experiment 5, Supplementary Table 1), with two of the overexpressed genes indirectly involved in regulation of the methanogenic acetyl-CoA pathway (Extended Data Fig. 3). These include methyl-COM reductase system component A2 (*atw*A), which is an ATP-binding protein^81^, and an NADPH-dependent F420 reductase (*npd*G). Despite *M. jannaschii* being a hyperthermophile, the number of overexpressed acetyl-CoA pathway-associated genes at high temperature (*n* = 2) was lower compared to the iron-sulfide experiments (*n* = 11). This indicates that the iron-sulfide chemical garden environment had a larger positive influence on the activity of the H_2_-dependent acetyl-CoA pathway metabolism relative to temperature.

While the genes encoding the acetyl-CoA pathway in the sedimentary iron-sulfide chemical garden were overexpressed relative to MMC medium, the growth rate of *M. jannaschii* was lower in the chemical gardens by comparison (Fig. 3a). The slower growth rate could be explained by the presence of additional bicarbonate (the carbon source), micronutrients, vitamins and trace metals supplied in the MMC medium, and excluded in the iron-sulfide chemical gardens (which only received FeCl_2_ and Na_2_S).

To conclude, the physiology of many methanogenic archaea is defined by H_2_-dependent syntrophy, where H_2_ is produced by a partner organism^82^. This relationship, whereby both partner organisms live together in a metabolic partnership, is considered to have an ancient origin^83^. Our results suggest, that on the early Eoarchaean Earth abiotic iron-sulfide geochemistry could have replaced H_2_-producing syntrophic partner organisms and provide an abiotic H_2_ source for H_2_-dependent methanogenic archaea. This could have been an ancient precursor to modern syntrophic partnerships, whereby H_2_ is sourced from biological fermentations.

## Outlook

We provide experimental testing of a key aspect of hydrothermal and iron-sulfide-rich environments for the emergence of life, namely that abiotic H_2_ produced in a sedimentary iron-sulfide chemical garden could have provided sufficient energy to fuel the survival and growth of Archaea in an Eoarchaean hydrothermal environment. Our results show that abiotic H_2_ produced by iron-sulfide mineral redox reactions is sufficient to promote exponential chemolithoautotrophic growth of a hyperthermophilic methanogen under ferruginous conditions. This physiological response was explained by the overexpression of acetyl-CoA pathway-encoding genes by *M. jannaschii* in the sedimentary iron-sulfide chemical gardens and an exergonic CO_2_-fixation pathway that has been described as a ‘free lunch that you are paid to eat’^84^. Our findings provide experimental support for theories^36,85,86^ predicting that the extreme and energy-limited conditions of iron-sulfide-rich environments of the Eoarchaean would have promoted an acetyl-CoA pathway-based metabolism. Our study points to FeS–Fe_3_S_4_ chemical gardens as potential hatcheries of life, primordial environments that could theoretically support a continuous evolution of the first metabolizing cells, through the progenote, to a methanogen.

## Methods

The hyperthermophilic methanogen *M. jannaschii*^52^ (DSM strain 2661, German Collection of Microorganisms and Cell Cultures GmbH) was cultivated in an MMC growth medium^68^, which was prepared at the Institute of Microbiology and German Archaea Centre at the University of Regensburg (Supplementary Methods). The strain was recultivated from the Bacteria Bank Regensburg and adopted to the medium by at least two serial transfers in MMC. The stationary-phase cell cultures were used to inoculate the experiments that are part of this study.

In total, five different sets of experiments were executed (Supplementary Table 1). Throughout our paper we refer to these experiments as: experiment 1, chimney formation (Fig. 1a); experiment 2, abiotic H_2_ formation (Fig. 2a and Extended Data Fig. 2); experiment 3, stable isotope labelling (Fig. 2b,c); experiment 4, *M. jannaschii* colonization and growth curve (Figs. 1b and 3a); and experiment 5, transcriptomics (Figs. 3b and 4 and Extended Data Figs. 3–5) and mineralogical analysis (Fig. 1c–f and Extended Data Fig. 1).

A detailed chimney formation protocol for experiment 1 is in Supplementary Methods. In experiment 2 and 3 (Supplementary Table 1), gas measurements of ^13^CO_2_, ^13^CH_4_ and H_2_ were performed using a GC-MS QP2020 NX connected to a headspace autosampler (Shimadzu) (see Supplementary Methods for protocol).

In experiment 4 (Supplementary Table 1) we compared the growth of *M. jannaschii* in the sedimentary iron-sulfide chemical garden at 80 °C (experiment 4a) to its growth in MMC medium (experiment 4c) as a positive control. As an additional negative control, we also measured the growth of *M. jannaschii* in sterile water (experiment 4b). The sterile water negative control accounted for the dilution factor introduced in the chemical garden, making the presence of iron-sulfide the only difference between both experiments 4a and 4b. Using the chemical garden from experiment 4a we tested the colonization of *M. jannaschii* on the iron-sulfide particles (Fig. 1b). Cell counts and particle attachment were visualized using an inverted fluorescence microscope (Leica Thunder Imager DMi) based on autofluorescence of the coenzyme F420 present in *M. jannaschii*^87^.

In experiment 5 (Supplementary Table 1) we compared the transcriptomic response of *M. jannaschii* at 80 °C in the sedimentary iron-sulfide chemical garden (experiment 5a) to the gene expression in MMC medium (experiment 5c) as a positive control and sterile water (experiment 5b) as a negative control. Furthermore, we performed transcriptomes on stationary-phase cultures stored at 25 °C (experiment 5d) as an additional low temperature comparison. Similar to experiment 4 the sterile water negative control was used to account for the dilution factor introduced by inoculating the stationary-phase culture into the chemical garden, making the presence of iron-sulfide the only difference between experiments 5a and 5b.

In experiment 5, RNA was extracted using the Direct-zol RNA Microprep kit (ZYMO Research)^75^, with several changes to the protocol to improve RNA extraction from the chemical gardens. Phosphate was added to reduce adsorbtion of RNA to the iron-sulfide minerals and chloroform was added to improve RNA recovery (Supplementary Methods). Transcriptomes were prepared using the Revelo RNA-Seq kit (Tecan) and raw reads were mapped against the annotated genome of *M. jannaschii*^88^ using BLASTx with DIAMOND^89^ to measure gene expression levels.

The mineralogy of sedimentary iron-sulfide chemical gardens (experiment 5a, Supplementary Table 1) was analysed using Raman spectroscopy according to previously reported methods^47^ and SEM with an EDX detector (protocol in Supplementary Methods). Full details on transcriptome preparations, bioinformatic analysis, cell counts, gas analysis, Raman spectroscopy, EDX analysis and MMC medium preparations are provided in Supplementary Methods^90,91^.

### Reporting summary

Further information on research design is available in the Nature Portfolio Reporting Summary linked to this article.

## Supplementary information

Supplementary Information Supplementary Tables 1–4 and Methods.

Reporting Summary

## Data Availability

Transcriptome data have been deposited in the NCBI short read archive under Bioproject ID PRJNA1157004.

## Code availability

All code used in this study is available via GitHub at https://github.com/williamorsi/bioinformatics_code_orsi.
