# Supplementary material for: Simulated early Earth geochemistry fuels a hydrogen-dependent primordial metabolism
Source: Nat Ecol Evol. 2025 Apr 30;9(5):769–78. doi: 10.1038/s41559-025-02676-w (PMC12066356; doi:10.1038/s41559-025-02676-w)
Supplement: Supplementary file 1 — Supplementary Tables 1–4 and Methods. [file 41559_2025_2676_MOESM1_ESM.pdf]

nature ecology & evolution

Supplementary information https://doi.org/10.1038/s41559-025-02676-w

Simulated early Earth geochemistry fuels a hydrogen-dependent primordial metabolism

In the format provided by the authors and unedited

Supporting Information

Table of contents

- **Extended Data figure legends**
  - Extended Data Figure 1 legend
  - Extended Data Figure 2 legend
  - Extended Data Figure 3 legend
  - Extended Data Figure 4 legend
  - Extended Data Figure 5 legend
- **Supplemental Tables**
  - Supplemental Table 1
  - Supplemental Table 2
  - Supplemental Table 3
  - Supplemental Table 4
- **Supplemental Methods**

Extended Data figure legends

Extended Data 1 **Sedimentary iron-sulfide chemical garden precipitates align with magnetic rings on a ring magnet plate, showing the presence of the magnetic iron-sulfide mineral greigite (Fe_3S_4).**

Extended Data 2 **Abiotic H_2 produced in the 500 mM sedimentary iron-sulfide chemical gardens at 80 °C with and without *M. jannaschii*.** Error bars show standard error of the means across three biological replicates, P value is the result of a one-sided T-test.

Extended Data 3 **Volcano plot analysis of gene expression of *M. jannaschii* transcriptomes in MMC Medium at 25 and 80 °C (*Experiments 5c, d*, Supplemental Table 1).** Of all significant genes (*P*=0.05, one-sided t-test), 96 are overexpressed at 80 °C (dark blue, *Experiment 5c*) compared to 25 °C. 241 genes were down regulated at 80 °C compared to 25 °C (light blue, *Experiment 5d*). The vertical line separates expressed genes that either increased or decreased in rank in the MMC medium, the dotted horizontal line represents the *P*-value cutoff for determining statistical significance (one-sided T-test). Of all overexpressed genes, two are part of the methanogenic acetyl CoA pathway: methyl coenzyme M reductase system component A2 (*atw*A) and NADPH-dependent F420 reductase (*npd*G).

Extended Data 4 **Volcano plot analysis of gene expression of *M. jannaschii* transcriptomes in sterile water relative to MMC medium (*Experiments 5b, c*, Supplemental Table 1).** Of all significant genes (*P*=0.05, one-sided t-test), 59 are overexpressed in the sterile water controls (blue, *Experiment 5b*) compared to MMC medium (beige, *Experiment 5c*). Significantly overexpressed genes are listed in Supplemental Table 4. The full list of overexpressed genes is provided in Supplemental Table 4.

Extended Data 5 **Functional annotation of overexpressed genes. (*Experiments 5a-c,* Supplemental Table 1).** For gene analysis, the arCOGs (Archaeal Clusters of Orthologous Genes) database was used[79]. (a) Out of 34 overexpressed genes in the sedimentary iron-sulfide chemical garden relative to MMC medium

(Fig. 4a), the majority of genes has a function in metabolism (blue) or information, storage and processing (red), especially in coenzyme transport (H) and translation (J). (b) Out of 46 overexpressed genes in the sedimentary iron-sulfide chemical garden relative to sterile H_2O, most genes have a function in metabolism (blue), especially in energy production and conversion (C). Genes regulating cellular processes and signaling (green) make a proportion of 17 % of the overexpressed genes. (c) In the sterile H_2O controls relative to MMC medium, 59 genes were overexpressed, with 44 % having a metabolic function (blue).

Supplemental Table 1 **Overview of all experiments.**

**Experiments**	**1**	**2**	**3**	**4**			**5**			
				a	b	c	a	b	c	d
Temperature (°C)	25	80	80	80	80	80	80	80	80	25
Environment	Iron-sulfide chemical garden	Iron-sulfide chemical garden[6]	Iron-sulfide chemical garden[6]	Iron-sulfide chemical garden[6]	Sterile H_2O	MMC medium	Iron-sulfide chemical garden[6]	Sterile H_2O	MMC medium	MMC medium
Volume flask (mL)	20	20	20	9	9	9	9	9	9	9
Volume inoculation from *M. jannaschii* (mL)	-	0-0.1	0.1	1	1	1	1	1	1	1
$FeCl_2$ concentration (mM)	500	10-500	500	10	<0.05	<0.05	10	<0.05	<0.05	<0.05
Na_2S concentration (mM)	500	10-500	500	10	6.4	6.4	10	0.64	6.4	6.4
^{13}C labelled $NaHCO_3$ (mM)	-	-	10	-	-	-	-	-	-	-
Duration (h)	1	10	10	48	48	48	4	4	4	N/A*
Biological replicates	3	3	3	3	3	3	3	3	3	2
Technical replicates	0	0	0	0	0	0	3	0	2	1
Total replicates	3	3	3	3	3	3	6	3	5	3
Purpose of experiment	[1]Chimneys	[2]Abiotic H_2	[3]Stable isotope labelling	[4]*M. Jannaschii* colonization of iron-sulfide particles, growth curves			[5]Transcriptomes, mineralogical analysis			

* N/A = not applicable, since culture was stored at 25 °C

[1] Chimney formation in Figure 1a

[2] Abiotic H_2 formation results in Figure 2a and Extended Data 2

[3] Stable isotope labelling results in Figure 2b and c

[4] *M. jannaschii* colonization of iron-sulfide particles (Experiment 4a only) in Figure 1b, growth curves in Figure 3a

[5] Gene expression results in Figure 3b, 4 and Extended Data 3-5; Mineralogical analysis (Experiment 5a only) in Figure 1c-f and Extended Data 1

[6] Sedimentary iron-sulfide chemical gardens, not chimneys

Supplemental Table 2 **Overexpressed genes by *M. jannaschii* in the sedimentary iron-sulfide chemical garden transcriptomes relative to MMC medium controls at 80°C (*Experiments 5a, c*, Supplemental Table 1).**

Gene	Abbreviation	Fold change	*P*-value	arCOGs
CO dehydrogenase/CO-methylating acetyl-CoA synthase complex subunit beta	CODH/ACS	22.6357	0.0053	C
MULTISPECIES: TRAM domain-containing protein		8.2528	0.0388	O
AAA family ATPase		6.8750	0.0114	R
methyl coenzyme M reductase, subunit alpha	*mcr*A	6.1477	0.0120	H
tetrahydromethanopterin S-methyltransferase subunit A-H	*mtr*A-H	5.8586	0.0337	H
methyl coenzyme M reductase, subunit beta	*mcr*B	5.5085	0.0068	H
acetyl-CoA carboxylase biotin carboxylase subunit		4.4005	0.0098	H
methyl-coenzyme M reductase operon protein D	*mcr*D	3.8810	0.0258	H
reverse gyrase		3.7310	0.0114	L
ATP synthase subunit A, B, K		3.7041	0.0119	C
ribosome assembly factor SBDS		3.6607	0.0370	J
methyl coenzyme M reductase, subunit gamma	*mcr*G	3.6374	0.0399	H
ATP-grasp domain-containing protein		3.6121	0.0062	R
methyl-coenzyme M reductase I operon protein C	*mcr*C	3.1454	0.0472	H
MULTISPECIES: 50S ribosomal protein		3.1316	0.0380	J
formylmethanofuran dehydrogenase subunit A, B and C	*fmd*A,B,C	3.0799	0.0340	C
right-handed parallel beta-helix repeat-containing protein		3.0762	0.0219	S
heat shock protein HSP16.5		2.8879	0.0135	O
formylmethanofuran-tetrahydromethanopterin N-formyltransferase	*ftr*	2.8516	0.0309	S
Mrp/NBP35 family ATP-binding protein		2.7880	0.0003	P
TIGR00703 family protein		2.7626	0.0002	S
translation elongation factor EF-1 subunit alpha		2.6667	0.0385	J
valine--tRNA ligase		2.6145	0.0485	J
elongation factor EF-2		2.5806	0.0468	J
alanine--glyoxylate aminotransferase family protein		2.5531	0.0321	K
carbamoyltransferase (HypF)		2.5074	0.0006	S
intein-containing RctB family protein		2.1915	0.0002	L
acetyl-CoA decarbonylase/synthase complex subunit alpha, beta, gamma	ACDS	1.9634	0.0106	C
ketol-acid reductoisomerase		1.9494	0.0387	E
metal-dependent hydrolase		1.9004	0.0025	R
acetolactate synthase large and small subunit		1.4749	0.0334	E

NCS2 family permease		1.4373	0.0001	G
glutamine-hydrolyzing GMP synthase		1.2648	0.0412	F
Asp-tRNA_Asn_/Glu-tRNA_Gln_ amidotransferase subunit GatA, GatB, GatC		1.2483	0.0142	J

List of overexpressed genes in 80 °C sedimentary iron-sulfide chemical gardens relative to MMC medium controls (*Experiments 5a, c*). Highlighted rows are those involved in the methanogenesis pathway. The fold change and *P*-value are provided for all overexpressed genes. The last column shows the Archaeal Clusters of Orthologous Genes (arCOGs) used for functional annotation. Abbreviations are explained in Extended Data 5.

Supplemental Table 3 **Overexpressed genes by *M. jannaschii* in the sedimentary iron-sulfide chemical garden transcriptomes relative to sterile water controls at 80°C (*Experiments 5a, b*, Supplemental Table 1).**

**Gene**	**Abbreviation**	**Fold change**	***P*-value**	**arCOGs**
CO dehydrogenase/CO-methylating acetyl-CoA synthase complex subunit beta	CODH/ACS	11.6569	0.0014	C
TldD/PmbA family protein		9.2565	0.0068	O
tetrahydromethanopterin S-methyltransferase subunit A-H	*mtr*A-H	8.8136	0.0440	H
methyl coenzyme M reductase, subunit gamma	*mcr*G	8.6859	0.0016	H
ATP-binding protein		7.7826	0.0367	P
LAGLIDADG family homing endonuclease		6.8523	0.0435	L
CoB-CoM heterodisulfide reductase subunit B and C	*hdr*B,C	6.7670	0.0372	C
anaerobic ribonucleoside-triphosphate reductase (activating protein)		6.7143	0.0093	S
carbamoyltransferase (HypF)		6.4600	0.0000	S
intein-containing phosphoenolpyruvate synthase		5.7358	0.0135	G
ribosome assembly factor SBDS		5.3802	0.0408	J
heat shock protein HSP16.5		5.1752	0.0294	O
methyl coenzyme M reductase, subunit beta	*mcr*B	4.9837	0.0296	H
flagellin		4.3873	0.0286	N
MULTISPECIES: 50S ribosomal protein		4.1200	0.0448	J
acetyl-CoA decarbonylase/synthase complex subunit alpha, beta, gamma	ACDS	4.0767	0.0170	C
methyl coenzyme M reductase, subunit alpha	*mcr*A	3.9583	0.0294	H
elongation factor EF-2		3.8750	0.0348	J
type I-A CRISPR-associated protein		3.7813	0.0074	V

ABC transporter ATP-binding protein		3.7313	0.0342	P
DUF2226 domain-containing protein		3.6264	0.0055	S
DUF505 domain-containing protein		3.6202	0.0049	S
DEAD/DEAH box helicase family protein		3.4161	0.0196	V
Mrp/NBP35 family ATP-binding protein		3.4021	0.0450	P
V-type ATP synthase subunit C, D, E, I		3.3629	0.0084	C
4Fe-4S binding protein		3.2652	0.0392	C
ATP synthase subunit A, B, K		3.1818	0.0127	C
AAA family ATPase		3.1429	0.0303	V
formylmethanofuran dehydrogenase subunit A, B and C	*fmd*A,B,C	2.8228	0.0399	C
valine--tRNA ligase		2.6969	0.0142	J
PIP-CTERM sorting domain-containing protein		2.6038	0.0445	M
ATP-dependent Clp protease proteolytic subunit		2.4879	0.0139	O
radical SAM protein		2.4235	0.0340	R
right-handed parallel beta-helix repeat-containing protein		2.3799	0.0278	S
2-oxoacid:ferredoxin oxidoreductase subunit beta, gamma		2.3696	0.0083	C
intein-containing RctB family protein		2.3372	0.0261	L
pyridoxal phosphate-dependent aminotransferase		2.1407	0.0467	E
iron ABC transporter substrate-binding protein		1.9538	0.0436	R
roadblock/LC7 domain-containing protein		1.8843	0.0464	R
PFL family protein		1.7955	0.0219	S
CBS domain-containing protein		1.7917	0.0459	R
50S ribosomal protein		1.7857	0.0277	J
ATP-grasp domain-containing protein		1.7574	0.0077	R
acetolactate synthase large and small subunit		1.7489	0.0095	E
acetyl-CoA carboxylase biotin carboxylase subunit		1.6067	0.0298	H
glutamine-hydrolyzing GMP synthase		1.3956	0.0442	F

List of overexpressed genes in 80 °C sedimentary iron-sulfide chemical gardens relative to sterile water controls (*Experiments 5a, b*). Highlighted rows are those involved in the methanogenesis pathway. The fold change and *P*-value are provided for all overexpressed genes. The last column shows the Archaeal Clusters of Orthologous Genes (arCOGs) used for functional annotation. Abbreviations are explained in Extended Data 5.

Supplemental Table 4 **Overexpressed genes by *M. jannaschii* in the sterile water transcriptomes relative to MMC medium transcriptomes at 80 °C (*Experiments 5b, c*, Supplemental Table 1).**

Gene	Fold change	*P*-value	arCOGs
MULTISPECIES: TRAM domain-containing protein	3.8566	0.0001	O
histone family protein	3.2148	0.0007	R
ZPR1 zinc finger domain-containing protein	2.7823	0.0420	S
acetyl-CoA carboxylase biotin carboxylase subunit	2.7579	0.0010	H
ATP-grasp domain-containing protein	2.7451	0.0003	R
type II/IV secretion system ATPase subunit	2.6053	0.0000	N
Methyl coenzyme M reductase, subunit beta	2.5989	0.0119	H
ketol-acid reductoisomerase	2.5517	0.0312	E
LemA family protein	2.4621	0.0003	S
phosphate ABC transporter substrate-binding protein PstS	2.2303	0.0045	P
alanine--glyoxylate aminotransferase family protein	2.1902	0.0339	K
AAA family ATPase	2.1875	0.0152	V
6-carboxytetrahydropterin synthase	2.1722	0.0428	S
DNA repair and recombination protein RadA and RadB	2.1516	0.0063	L
DNA-directed DNA polymerase II large and small subunit	2.0978	0.0099	L
3-phosphoshikimate 1-carboxyvinyltransferase	2.0736	0.0089	S
pyruvate synthase subunit PorA, PorB, PorC, PorD	1.9880	0.0451	C
phosphoglycerate dehydrogenase	1.9873	0.0499	H
CO dehydrogenase/CO-methylating acetyl-CoA synthase complex subunit beta	1.9767	0.0007	C
heat shock protein HSP16.5	1.9684	0.0213	O
60S ribosomal export protein NMD3	1.9233	0.0116	J
winged helix-turn-helix transcriptional regulator	1.9217	0.0224	K
4-demethylwyosine synthase TYW1	1.8930	0.0076	J
MTH895/ArsE family thioredoxin-like protein	1.8858	0.0028	R
hydroxymethylglutaryl-CoA reductase _NADPH_	1.8780	0.0339	I
DNA topoisomerase IV subunit A	1.8734	0.0134	L
glutamate synthase-related protein	1.8668	0.0022	E
right-handed parallel beta-helix repeat-containing protein	1.8465	0.0149	S
DUF2540 domain-containing protein	1.7391	0.0464	S
formate dehydrogenase subunit alpha	1.7301	0.0017	C
type II glyceraldehyde-3-phosphate dehydrogenase	1.7032	0.0094	G
elongation factor 1-beta	1.6781	0.0188	K
phosphoadenosine phosphosulfate reductase family protein	1.6752	0.0380	E
HsdR family type I site-specific deoxyribonuclease	1.6620	0.0025	L
dihydroneopterin 2,3-cyclic phosphate phosphodiesterase	1.6383	0.0118	R
adenylosuccinate lyase	1.6221	0.0115	F
precorrin-3B C_17_-methyltransferase	1.6174	0.0268	H
methyl-coenzyme M reductase glutamine C-methyltransferase	1.6007	0.0500	H
DNA polymerase domain-containing protein	1.5897	0.0361	L
cobalt transporter CbiM	1.5730	0.0443	P
IS607 family transposase	1.5709	0.0478	X
minichromosome maintenance protein MCM	1.5599	0.0184	L
S-methyl-5-thioribose-1-phosphate isomerase	1.5487	0.0119	E
UPF0058 family protein	1.5446	0.0026	S

chloride channel protein	1.5378	0.0190	P
hydroxylamine reductase	1.5304	0.0088	C
magnesium/cobalt transporter CorA	1.4936	0.0387	P
gamma carbonic anhydrase family protein	1.4924	0.0430	P
geranylgeranylglyceryl/heptaprenylglyceryl phosphate synthase	1.4733	0.0385	I
FAD-dependent oxidoreductase	1.4683	0.0425	R
arginine--tRNA ligase	1.4530	0.0234	J
sodium:proton antiporter	1.4264	0.0449	P
DUF5814 domain-containing protein	1.4222	0.0046	S
DUF128 domain-containing protein	1.3762	0.0401	S
_5-formylfuran-3-yl_methyl phosphate synthase	1.3758	0.0300	R
DASS family sodium-coupled anion symporter	1.2249	0.0354	P
tRNA pseudouridine_38-40_ synthase TruA	1.1991	0.0249	J
cation:proton antiporter subunit C	1.1931	0.0334	P
pantoate kinase	1.0840	0.0422	H

List of overexpressed genes in 80 °C sterile water controls relative to MMC medium controls. The fold change and *P*-value are provided for all overexpressed genes. The last column shows the Archaeal Clusters of Orthologous Genes (arCOGs) used for functional annotation.

Supplemental Methods

MMC medium preparation and cell cultivation

The MMC medium was prepared as described previously[68], which is one of the optimal growth media (pH=6.5) used for *Methanocaldococcus jannaschii* cultivation. 20 mL of the anoxic medium was added to 125 mL glass serum bottles inside an anoxic chamber, crimp-sealed and overpressured with 2 bars of a 20:80 H_2/CO_2 gas mix, and autoclaved for 20 min at 121 °C. Cells were grown by adding 200 µL of a *M. jannaschii* stock to a serum bottle containing 20 mL of sterile MMC medium. Transfer was done using a 1 mL luer syringe (ERSTA) and a 23Gx1 ¼ needle through a gas tight rubber stopper (Henke Sass Wolf). After inoculation, the serum bottles were incubated at 85 °C on a shaker table for 24 hours. Full-grown cultures were stored at room temperature and used as a starting inoculum for the sedimentary iron-sulfide chemical garden experiments and associated controls.

Overview of experimental set ups and controls

In total, five different sets of experiments were executed which are described in Supplemental Table 1. Throughout our manuscript we refer to these experiments as: *Experiment 1* (Fig. 1a), *Experiment 2* (Fig. 2a and Extended Data 2), *Experiment 3* (Fig. 2b and c), *Experiment 4* (Fig. 1b and 3a) and *Experiment 5* (Fig. 1c-f, Fig. 3b, Fig. 4 and Extended Data 1, 3-5). Below, we provide a detailed description of each experiment and its purpose.

Experiment 1 - Iron-sulfide chimneys

The setup for creating chimney structures (*Experiment 1*, Supplemental Table 1) was slightly modified from a previous chimney formation protocol[47]. We performed chimney growth experiments (n=3) using a single-syringe pump (Cole Parmer, Model 100 78-9100C) that contained a 10 ml Luer-lock plastic syringe (B Braun) and a sterican hypodermic-needle (B Braun, 1.20x50 mm, 18 G x 2). The syringe was filled with an acidic sulfidic solution made with 0.5 M Na_2S (90+% pure, Thermo Scientific, pH=3, titrated with 1 M HCl) and sterile MilliQ water. We used this setup, because ancient geological formations show the presence of hydrothermal iron-sulfide minerals in temporal proximity to some of the earliest signs of life in the fossil record[8,9,16].

The syringe pump and the syringe were vertically oriented beneath a 20 mL inverted crimp-sealed glass vial, where the bottom had been cut off and the opening was sealed with parafilm. The sulfidic solution was injected into the crimped vial containing a sterile filtered (PES (25 mm, 0.22 µm) syringe filter (VWR)) ferruginous 0.5 M $FeCl_2$ (Roth, pH=6) solution, that was constantly bubbled with pure N_2 (99.999 %, Linde) to ensure anoxic conditions. The flow rate of the injection was 5 ml/h. We decided to use this flow rate because it produced a stable chimney structure in contrast to faster flow rates that caused a faster chimney growth, but less stable structures.

Compared to the former chimney formation protocol[47], the syringe needle was now directly injected into the glass vial without any connecting tubing.

Images of the initial chimney growth phase were taken one minute, 5 minutes and 10 minutes after the injection and again at 60 minutes, where the maximum growth was observed. For imaging, a Sony Alpha a6000 mirrorless camera and a 50 mm Sony macro lens and a tripod were used. Each image was processed in Adobe Lightroom and the average height of the chimneys (n=3) after 10 and 60 minutes was determined in Adobe Photoshop with the ruler tool by comparing the chimney size to the standard size of the aluminum crimp seal (2 cm).

Chimney formation at high temperature was not possible due to gas bubbles forming when heating the sulfidic solution in the syringe to 80 °C. In order to observe stable chimney structures, we injected the sulfidic solution at room temperature, which ensured a stable chimney that could be photographed. Immediately after the injection of the sulfidic solution, a black precipitate formed at the tip of the syringe needle that grew and formed a chimney structure in 5 to 10 minutes (Fig. 1a).

The salinity of the Archaean Ocean is poorly constrained, but likely contained Na and Cl 1.5 - 2x the salinity of modern marine conditions[51] and extremely low concentrations of sulfate[49]. Our experiments attempted to reflect these conditions in that they contained Na and Cl at levels slightly higher than modern ocean salinities, specifically, 22 g/kg of Na in the fluid and 35 g/kg of Cl in the ferruginous solution. Sulfate was absent in our experimental setup.

Experiment 2 - Abiotic H_2 production in the sedimentary iron-sulfide chemical garden

Abiotic hydrogen (H_2) formation was tested in the sedimentary iron-sulfide chemical gardens at 80 °C (*Experiment 2*, Supplemental Table 1). Three replicate experiments in crimp-sealed 20 mL glass vials (VWR) were performed using sterile milliQ water with $FeCl_2$ (Roth) and Na_2S (Roth) concentrations ranging from 10 mM, 20 mM, 100 mM and 500 mM. The $FeCl_2$ solution was sterile filtered with a PES (25 mm, 0.22 µm) syringe filter (VWR) to remove precipitated iron-oxide

particles (and any other contaminating microbial cells) and maintain a ferruginous solution primarily of Fe(II). Before crimping the vials, the Na_2S was added, which immediately resulted in a black color change indicating the rapid precipitation of iron-sulfide minerals. The vials were immediately crimped and bubbled with pure N_2 (Linde) for at least 1 min to remove remaining oxygen traces in the vial. For the duration of the experiment, the crimped vials were kept on a preheated ThermoMixer C (Eppendorf) at 80 °C and incubated for 10 hours. Testing over a range of Fe and S concentrations showed whether the production of abiotic H_2 is dependent on the Fe(II) and Na_2S concentration alone (Fig. 2a). A set of sedimentary iron-sulfide chemical garden experiments with 0.5 M Fe(II) and Na_2S were conducted with, and without, the addition of *M. jannaschii* culture to investigate, whether the methanogens could consume the abiotic H_2 under the extreme conditions and without additional nutrients (Extended Data 2).

Experiment 3 - Stable isotope labeling experiment

To track the production and consumption of H_2, CO_2, and CH_4 in the 80 °C sedimentary iron-sulfide chemical gardens in the presence of *M. jannaschii,* three replicate chemical garden experiments were prepared in 20 mL headspace vials (VWR), containing 0.5 M $FeCl_2$ (Roth) and Na_2S (Roth), 10 mM ^{13}C-labeled $NaHCO_3$ (Sigma Aldrich) and 0.1 mL of stock culture of *M. jannaschii (Experiment 3,* Supplemental Table 1, Fig. 2c*)* in sterile milliQ water. The $FeCl_2$ solution was sterile filtered and the crimped flasks were bubbled with N_2. The experiments (n=3) were performed at 80 °C on a preheated ThermoMixer C (Eppendorf) and incubated for 10 hours.

Experiments 2 and 3: GC-MS analysis

Liquid samples were taken at the end of *Experiment 2* and every hour over the experimental duration of 10 hours for *Experiment 3*. Specifically, the flasks were inverted and 0.2 mL of incubation material (iron-sulfide slurries) was transferred to another 20 mL gas tight headspace

vials that were crimp sealed with butyl stoppers. The crimp sealed flasks were inverted and incubated at room temperature for 24 hours. From those flasks, the headspace gas concentrations of H_2, CO_2, and CH_4 and ^{13}C-labeling in CO_2, and CH_4 was determined using gas chromatography mass spectrometry (GC-MS). The samples were heated to 60 °C for 5 minutes in a headspace sampler, and 1 ml of headspace gas was sampled via a headspace autosampler connected to a gas chromatograph with a quadropole mass spectrometer as the detector (GCMS-QP2020 NX, Shimadzu). Helium was used as the carrier gas. This GC-MS setup was calibrated for H_2, CO_2, and CH_4 using a pre-separation column [U-Bond, 0.32 mm ID, 10 µm Film, 30 m] to separate larger molecules, a pre-split to separate corrosive H_2S from the chemical garden experiment, followed by a second column [Carboxen-1010 Plot, 0.32 mm ID, 15 µm Film, 30 m] for separating H_2, CO_2, and CH_4. The elution times for H_2, CO_2, and CH_4 were determined by comparison to analytical standards of highly pure (99.999%) gases (Linde). The headspace concentrations of the gases were quantified by comparing them against standard curves of a gas dilution series, and the dissolved concentrations of gases were calculated using Henry's Law (Fig. 2a and Extended Data 2).

The ^{13}C-labeling in CO_2 and CH_4 was taken as percentage of ^{13}C-labeled CO_2 (m/z=45) or ^{13}C-labeled CH_4 (m/z=17) relative to the unlabeled CO_2 (m/z=44) or unlabeled CH_4 (m/z=16). These percentages were calculated via integration of the respective peak areas in the GC-MS trace from labeled and unlabeled CH_4 and CO_2. All experimental treatments (that had added ^{13}C-bicarbonate) had $^{13}CO_2$ and $^{13}CH_4$ percentages higher than the corresponding unlabeled control flasks, which consistently reflected the natural abundance of ^{13}C in the environment, which is 1.1 % (SD = 0.05).

Experiment 4 - M. jannaschii growth in high temperature sedimentary iron-sulfide chemical gardens

High-temperature (80 °C) sedimentary iron-sulfide chemical gardens for testing the growth of *M. jannaschii* were prepared in 10 mL sterile crimped glass vials (VWR) containing 8 mL of a ferruginous solution of 10 mM $FeCl_2$ (Roth, pH 4.5) (*Experiment 4a, Supplemental Table 1*) and sterile milliQ water. This solution was sterile filtered with a PES (25 mm, 0.22 μm) syringe filter (VWR) to remove precipitated iron-oxide particles (and any other contaminating microbial cells) and maintain a ferruginous solution primarily of Fe(II). Before crimping the vials, 10 mM Na_2S (90+% pure, pH=11, Thermo Scientific) was added, which immediately resulted in a black color change indicating the rapid precipitation of iron-sulfide particles. The vials were immediately crimped and bubbled with pure N_2 (Linde) for at least 1 min to remove remaining oxygen traces in the vial. The pH of the iron-sulfide chemical gardens was 5.5. In addition to the chemical gardens, two controls with sterile water and MMC medium were performed (*Experiments 4b and 4c, Supplemental Table 1*). The sterile water control accounted for the dilution factor introduced in the iron-sulfide chemical garden, making the presence of iron-sulfide the only difference between *Experiments 4a* and *4b (Supplemental Table 1)*. All three sedimentary iron-sulfide chemical gardens (*Experiments 4a, 4b, 4c*) were prepared at room temperature and inoculated with 1 mL of a stationary phase *M. jannaschii* culture using a 1 mL luer syringe (ERSTA) and a 23Gx1 ¼ luer lock needle (Henke Sass Wolf) leaving no headspace. No headspace was applied, because abiotic H_2 was only detectable in the sedimentary iron-sulfide chemical gardens when a headspace was not present. After inoculation, the crimp-sealed vials containing the *M. jannaschii* cells were placed in a preheated ThermoMixer C (Eppendorf) at 80 °C and incubated for 48 hours. The 48 hours incubation was required for the cultures to reach an exponential phase (which occurred after 24 hours).

In all treatments (*Experiment 4, Supplemental Table 1*), a 50 μL sample was taken at every time point, every hour in the first 8 hours, every 2 hours until hour 30 and every 6 hours until hour 48. Cell counts were performed with the inverted fluorescence microscope (Leica Thunder Imager DMi). From each 50 μL sample, 3 images were taken with the microscope, making 9 images per

treatment and per time point. In order to prepare the samples for microscopy, the 50 µL drop was mixed with 10 µL of 2 % ROTI Histofix (Roth) for cell preservation and 10 µL of ROTI Mount FluorCare (Roth) water soluble mounting media before adding the drop to a microscope slide. After adding the cover slip, the drop was equally distributed below the cover slip, which had an area of 1140 mm^2. Slides were stored in the dark at 4 °C.

Cell counts were done using the DAPI LED 405 Filter (Leica), the 40x objective and the Las X software. In total, 594 images were taken (22 time points, 9 images per time point, 3 different treatments). Each image had an area of 0.1089 mm^2. Cells were counted using the ImageJ software. Growth rates were calculated according to Widdel[90]. The effects of the hydrothermal iron-sulfide chemical gardens on *M. jannaschii* growth over 48 hours are displayed in Fig. 3a. Colonization of iron-sulfide particles after 48 hours (*Experiment 4a* only, Fig. 1b) was visualized with the inverted fluorescence microscope as well.

Experiment 5 - M. jannaschii gene expression and mineralogical analysis of iron-sulfide

For transcriptomic gene expression analysis, a 1 mL *M. jannaschii* culture was added to the high-temperature (80 °C) sedimentary iron-sulfide chemical gardens (pH 5.5) (*Experiment 5a*, Supplemental Table 1) that were prepared like the chemical gardens in *Experiment 4a*. Two 80 °C control experiments were performed with sterile water and MMC medium (*Experiments 5b and 5c*). After inoculation, the crimp-sealed vials containing the *M. jannaschii* cells were placed in a preheated ThermoMixer C (Eppendorf) at 80 °C and incubated for 4 hours. Additionally, we performed transcriptomes on stationary phase cultures stored at 25 °C (*Experiment 5d*) as an additional low temperature comparison.

Experiment 5 - RNA extraction and library preparation

RNA was extracted from *Experiments 5a-d* (Supplemental Table 1). RNA extraction of *M. jannaschii* proceeded immediately after the 4 hours were over. Vials were taken out of the 80 °C ThermoMixer and cooled to room temperature for a few minutes. Vials were uncrimped and the entire content (9 mL) was transferred to a 15 mL tube (Sarstedt). For RNA extraction, the Direct-zol RNA Microprep kit (ZYMO Research) and the protocol from Grünberger et al.[75] was used, but the protocol was modified in some steps to account for the large sample size. Samples were centrifuged for 10 min at 4255 x g in an Allegra X-30R centrifuge (Beckman Coultier). The supernatant was discarded. Then 2 mL of TRI Reagent (ZYMO Research) and 0.5 M Na_2HPO_4 powder was added to each tube. Na_2HPO_4 has been shown to help detach RNA from iron-rich samples in similar experiments[47]. Tubes were vortexed and incubated at room temperature for 10 min, then centrifuged again for 10 min at 4255 x g. The supernatant was transferred to a 2 mL LoBind tube (Eppendorf), where 10 µL Proteinase K (Merck) and 200 µl Chloroform p.A. (Roth) were added. Tubes were shaken for 15 seconds, incubated at room temperature for 10 min, then centrifuged for 20 min at 4 °C at 12000 x g. The upper aqueous phase was transferred to a 1.5 mL LoBind tube (Eppendorf). RNA binding and elution was done as described in the Direct-zol RNA Microprep kit. But, the DNAse I mix was prepared differently. 5 µL DNAse I and 75 µL DNase I digestion buffer were mixed and spin columns were incubated for 30 minutes at room temperature. RNA wash proceeded as described in the Microprep kit. For RNA elution, 20 µL of nuclease-free water was added to the spin column membrane. For RNA quality control, the Qubit 3.0 Fluorometer (life technologies) and the RNA High Sensitivity assay kit (Thermo Fisher Scientific) were used. The RNA concentration from the 80 °C sterile water control (*Experiment 5b*) and 10mM iron sulfide chemical garden (*Experiment 5a*) were 2.52 ng/µL, 2.90 ng/µL respectively. The RNA extracted from the exponentially growing cells in the MMC medium control (*Experiment 5c*) was too high to be initially measured with the Qubit High Sensitivity kit (above quantification range in the Qubit kit). Therefore, we diluted the RNA from the positive control to a concentration of 10 ng/µL prior to making the transcriptome libraries. We only measured RNA

yields prior to transcriptome library preparation in one of all biological replicates each in order to reduce the amount of time between extracting the RNA and transcriptome library preparation, because RNA degrades very quickly. After successful RNA extraction, library preparation proceeded immediately without freezing the samples.

RNA-Seq libraries were prepared using the Revelo RNA-Seq High Sensitivity Kit. The protocol used various thermal cycler programs that were done in a Mastercycler Nexus X2 (Nexus). Library amplification was done using 7 to 8 PCR cycles. Library quantification was done using the 2100 Bioanalyzer (Agilent) and the High Sensitivity DNA kit (Agilent). If necessary, samples were diluted to a final molarity of 2 to 10 nM and shipped to Procomcure GmbH in Salzburg, Austria for Illumina Sequencing.

Experiment 5 - Bioinformatic and statistical analyses

Transcriptome RNA-Seq libraries were quality controlled using CLC Genomics Workbench as described previously[91] and raw reads were mapped against the annotated genome of *M. jannaschii*[88] using BLASTx with DIAMOND[89] to measure gene expression levels. Genes were grouped based on descriptions provided in the annotation, for example duplicate genes in the genome with the same annotation. The genes were ranked in order from the most expressed to least expressed, based on read mappings in each experiment. Genes that were only detected in a single replicate were not considered in the rankings. Genes that were detected in two or more transcriptome replicates had their ranks averaged. The averages of the expressed gene ranks between replicate transcriptomes for each experiment were used to calculate the fold change in expressed gene rank per experimental treatment ($\log_2$). The adjusted *P*-value ($-\log_{10}$) was calculated with a one-sided t-test, using all replicate transcriptomes from each experimental treatment.

Experiment 5 - Raman Spectroscopy and Scanning Electron Microscopy of iron-sulfide minerals

Mineralogical analysis of the formed iron-sulfide phases in the sedimentary chemical gardens (*Experiment 5a*, Supplemental Table 1) was done using the Raman spectrometer (HORIBA Jobin Yvon XploRA, Mineral State Collection Munich). After the 4-hour experiment on the ThermoMixer at 80 °C, a 50 µl sample drop was taken from the uncrimped vial and placed on a microscope slide. The drop was dried at 50 °C in the Bambino hybridization oven. The dried sample was stored in a purged 50 mL tube (Sarstedt) to avoid rapid oxidation. All spectra were collected using the same settings: the green laser with a wavelength of 532 nm, the ×100 long working distance (LWD) objective (0.80 numerical aperture), a spectral range from 50 to 4000 cm^{-1}, a 10 s acquisition time, 2 accumulations, 100 µm confocal hole and 50 µm slit, 1800 grooves/mm grating and a filter with 10 % optical density of the incoming laser to avoid laser-induced oxidation of the dried sample. The Labspec 6 software was used to process the acquired spectra and the database from the RRUFF™ Project[57] was used for the analysis. The instrument was calibrated using a silicon wafer (520.7 cm^{-1}) every day and immediately before measuring.

Dried iron-sulfide from the sedimentary chemical gardens (*Experiment 5a*) was analysed in a scanning electron microscope (SEM) (Zeiss DSM 960 A). The sample was kept under an inert atmosphere (N_2) until placed in the vacuum chamber of the SEM. All measurements were carried out using an acceleration voltage of 20 kV, a beam current of 60 µA and a vacuum pressure of 10^{-6} hPa. Elemental analysis of the iron-sulfide sample was carried out with the electron dispersive X-ray spectroscopy detector (EDX).
