# Supplementary material for: Simulated early Earth geochemistry fuels a hydrogen-dependent primordial metabolism
Source: Nat Ecol Evol. 2025 Apr 30;9(5):769–78. doi: 10.1038/s41559-025-02676-w (PMC12066356; doi:10.1038/s41559-025-02676-w)
Supplement: Supplementary file 2 — Reporting Summary [file 41559_2025_2676_MOESM2_ESM.pdf]

nature portfolio

Corresponding author(s): William Orsi

Last updated by author(s): Nov 13, 2024

Reporting Summary

Nature Portfolio wishes to improve the reproducibility of the work that we publish. This form provides structure for consistency and transparency in reporting. For further information on Nature Portfolio policies, see our Editorial Policies and the Editorial Policy Checklist.

Statistics

For all statistical analyses, confirm that the following items are present in the figure legend, table legend, main text, or Methods section.

n/a	Confirmed	
☐	☒	The exact sample size (*n*) for each experimental group/condition, given as a discrete number and unit of measurement
☐	☒	A statement on whether measurements were taken from distinct samples or whether the same sample was measured repeatedly
☐	☒	The statistical test(s) used AND whether they are one- or two-sided<br>*Only common tests should be described solely by name; describe more complex techniques in the Methods section.*
☒	☐	A description of all covariates tested
☒	☐	A description of any assumptions or corrections, such as tests of normality and adjustment for multiple comparisons
☐	☒	A full description of the statistical parameters including central tendency (e.g. means) or other basic estimates (e.g. regression coefficient) AND variation (e.g. standard deviation) or associated estimates of uncertainty (e.g. confidence intervals)
☐	☒	For null hypothesis testing, the test statistic (e.g. *F*, *t*, *r*) with confidence intervals, effect sizes, degrees of freedom and *P* value noted<br>*Give P values as exact values whenever suitable.*
☒	☐	For Bayesian analysis, information on the choice of priors and Markov chain Monte Carlo settings
☒	☐	For hierarchical and complex designs, identification of the appropriate level for tests and full reporting of outcomes
☒	☐	Estimates of effect sizes (e.g. Cohen's *d*, Pearson's *r*), indicating how they were calculated

Our web collection on statistics for biologists contains articles on many of the points above.

Software and code

Policy information about availability of computer code

Data collection	Illumina software was used to demultiplex transcriptomes sequenced on the Illumina MiniSeq platform. CLC Genomics Workbench (version 9.5.4) was used to convert fastq files to fasta format, for the transcriptomes.
Data analysis	R was used to perform Principal Component Analysis and Analysis of Similarity (ANOSIM) statistical tests. DIAMOND was used to perform BLASTx searches of transcriptome reads (unassembled) against the M. jannaschii reference genome. A custom python script was used to summarize the BLASTx results per sample per gene in the reference genome and is available freely online through the LMU Open Science website (the URL is provided in the Methods of the paper). To analyze the RAMAN data and process the spectra, Labspec6 software was used. The cell counts were made using Las X software (part of the Leica inverted microscope) and ImageJ software. The stable isotope labeling data for gases was analyzed using the software pre-installed on the GCMS-QP2020 NX intrument.

For manuscripts utilizing custom algorithms or software that are central to the research but not yet described in published literature, software must be made available to editors and reviewers. We strongly encourage code deposition in a community repository (e.g. GitHub). See the Nature Portfolio guidelines for submitting code & software for further information.

Data

Policy information about availability of data

All manuscripts must include a data availability statement. This statement should provide the following information, where applicable:

- Accession codes, unique identifiers, or web links for publicly available datasets
- A description of any restrictions on data availability
- For clinical datasets or third party data, please ensure that the statement adheres to our policy

Transcriptome data have been deposited in the NCBI short read archive under Bioproject ID: PRJNA1157004

Research involving human participants, their data, or biological material

Policy information about studies with human participants or human data. See also policy information about sex, gender (identity/presentation), and sexual orientation and race, ethnicity and racism.

Reporting on sex and gender	Not applicable.
Reporting on race, ethnicity, or other socially relevant groupings	Not applicable.
Population characteristics	Not applicable.
Recruitment	Not applicable.
Ethics oversight	Not applicable.

Note that full information on the approval of the study protocol must also be provided in the manuscript.

Field-specific reporting

Please select the one below that is the best fit for your research. If you are not sure, read the appropriate sections before making your selection.

☐ Life sciences ☐ Behavioural & social sciences ☒ Ecological, evolutionary & environmental sciences

For a reference copy of the document with all sections, see nature.com/documents/nr-reporting-summary-flat.pdf

Ecological, evolutionary & environmental sciences study design

All studies must disclose on these points even when the disclosure is negative.

Study description	In total, five different sets of experiments were executed (see Supplemental Table 1). Throughout our manuscript we refer to these experiments as: Experiment 1 - chimney formation (Fig. 1a), Experiment 2 - abiotic H2 formation (Fig. 2a, Extended Data 3), Experiment 3 - stable isotope labeling (Fig. 2b, Extended Data 2), Experiment 4 - M. jannaschii colonization and growth curve (Fig. 1b and 3a) and Experiment 5 - transcriptomics (Fig. 3b, 4 and Extended Data 4) and mineralogical analysis (Fig. 1c-f).
Research sample	The hyperthermophilic methanogen Methanocaldococcus jannaschii was cultivated in a MMC growth medium, which was prepared at the Institute of Microbiology and German Archaea Centre at the University of Regensburg (see Supplemental Methods). The stationary phase cell cultures were used to inoculate the experiments that are part of this study.
Sampling strategy	A detailed chimney formation protocol for Experiment 1 is displayed in the SI Methods. In Experiment 2 and 3 (Supplemental Table 1) gas measurements of 13CO2, 13CH4, and H2 were performed using a GC-MS QP2020 NX connected to a headspace autosampler (Shimadzu) (see Supplemental Methods for protocol).<br>In Experiment 4 (Supplemental Table 1) we compared the growth of M. jannaschii in the iron-sulfide chemical garden at 80 °C (Experiment 4a) to its growth in MMC medium (Experiment 4c) as a positive control. As an additional negative control, we also measured the growth of M. jannaschii in sterile water (Experiment 4b). The sterile water negative control accounted for the dilution factor introduced in the iron-sulfide chemical garden, making the presence of iron-sulfide the only difference between both Experiments 4a and 4b. Using the chemical garden from Experiment 4a we tested the colonization of M. jannaschii on the iron–sulfide particles (Fig. 1b). Cell counts and particle attachment were visualized using an inverted fluorescence microscope (Leica Thunder Imager DMi) based on autofluorescence of the coenzyme F420 present in M. jannaschii.<br>In Experiment 5 (Supplemental Table 1) we compared the transcriptomic response of M. jannaschii at 80 °C in the iron-sulfide chemical garden (Experiment 5a) to the gene expression in MMC medium (Experiment 5c) as a positive control and sterile water (Experiment 5b) as a negative control. Furthermore, we performed transcriptomes on stationary phase cultures stored at 25 °C (Experiment 5d) as an additional low temperature comparison. Similar to Experiment 4 the sterile water negative control was used to account for the dilution factor introduced by inoculating the stationary phase culture into the iron-sulfide chemical garden, making the presence of iron-sulfide the only difference between Experiments 5a and 5b.<br>In Experiment 5 RNA was extracted using the Direct-zol RNA Microprep kit (ZYMO Research), with several changes to the protocol to

	improve RNA extraction from the iron-sulfide chemical gardens. Phosphate was added to reduce adsorbtion of RNA to the iron-sulfide minerals, and chloroform was added to improve RNA recovery (see Supplemental Methods). Transcriptomes were prepared using the Revelo RNA-Seq kit (Tecan) and raw reads were mapped against the annotated genome of M. jannaschii using BLASTx with DIAMOND to measure gene expression levels. The mineralogy of iron-sulfide chemical gardens (Experiment 5a, Supplemental Table 1) was analyzed using Raman spectroscopy as described previously77 and scanning electron microscopy (SEM) with an EDX detector (protocol in SI Methods). Full details on transcriptome preparations, bioinformatic analysis, cell counts, gas analysis, Raman spectroscopy, EDX analysis and MMC medium preparations are provided in the Supplemental Methods.
Data collection	Data was collected either immediately after each experiment by Vanessa Helmbrecht, with the exception of transcriptome samples , EDX, and RAMAN analyses. EDX for elemental analysis was performed within 24 hours. For transcriptomes, the RNA was converted to cDNA libraries and sequenced within 2 to 3 weeks. For the RAMAN analyses, the measurements were made within 24 hours. The cell count data was collected within hourly intervals during growth experiment.
Timing and spatial scale	The cell count data was collected within hourly intervals during growth experiment that lasted 48 hours. The transcriptome data was collected after 4 hours of incubations. The RAMAN data from the minerals was collected after an 10 hour experiment. The stable isotope probing gas data was sampled every 2 hours over a 10 hour time interval. Chimney growth experiments lasted 1 hour.
Data exclusions	No data or samples were excluded.
Reproducibility	Biological replicates (n=3) were performed for all experiments.
Randomization	No randomization was performed, samples were grouped into biological replicates based on controlled conditions compared to a single factor that differed between the control and experiment.
Blinding	No blinding was performed.

Did the study involve field work? ☐ Yes ☒ No

Reporting for specific materials, systems and methods

We require information from authors about some types of materials, experimental systems and methods used in many studies. Here, indicate whether each material, system or method listed is relevant to your study. If you are not sure if a list item applies to your research, read the appropriate section before selecting a response.

Materials & experimental systems

n/a	Involved in the study
☒	☐ Antibodies
☒	☐ Eukaryotic cell lines
☒	☐ Palaeontology and archaeology
☒	☐ Animals and other organisms
☒	☐ Clinical data
☒	☐ Dual use research of concern
☒	☐ Plants

Methods

n/a	Involved in the study
☒	☐ ChIP-seq
☒	☐ Flow cytometry
☒	☐ MRI-based neuroimaging

Plants

Seed stocks	No plants were used.
Novel plant genotypes	No plants were used.
Authentication	No plants were used.
